# The Impact of Operational Parameters on Polypropylene Membrane Performance during the Separation of Oily Saline Wastewaters by the Membrane Distillation Process

**DOI:** 10.3390/membranes12040351

**Published:** 2022-03-22

**Authors:** Wirginia Tomczak, Marek Gryta

**Affiliations:** 1 Faculty of Chemical Technology and Engineering, Bydgoszcz University of Science and Technology, 3 Seminaryjna Street, 85-326 Bydgoszcz, Poland; 2 Faculty of Chemical Technology and Engineering, West Pomeranian University of Technology in Szczecin, ul. Pułaskiego 10, 70-322 Szczecin, Poland

**Keywords:** direct contact membrane distillation, flow velocity, fouling, long-term performance, oily saline wastewater, polypropylene membrane, process parameters, scaling, temperature, wetting

## Abstract

In the present study, membrane distillation (MD) was applied for the treatment of oily saline wastewaters produced on ships sailing the Baltic Sea. For comparison purposes, experiments were also carried out with model NaCl solutions, the Baltic Seawater and oil in water emulsions. The commercial Accurel PP V8/2 membranes (Membrana GmbH, Germany) were used. In order to investigate the impact of the operational parameters on the process performance, the experiments were conducted under various values of the feed flow velocity (from 0.03 to 0.12 m/s) and the feed temperature (from 323 to 343 K). The obtained results highlight the potential of PP membranes application for a stable and reliable long-term treatment of oily wastewater. It was demonstrated that the permeate flux increased significantly with increasing feed temperature. However, the lower temperature ensured the limited scaling phenomenon during the treatment of oily wastewaters. Likewise, increasing the feed flow velocity was beneficial to the increase in the flux. Moreover, it was found that performing a cyclic rinsing of the module with a 3% HCl solution is an effective method to maintain a satisfactory module performance. The present study sheds light on improving the MD for the treatment of oily wastewaters.

## 1. Introduction

Membrane distillation (MD) is a separation technology driven by transmembrane vapor pressure difference. The microporous membrane acts as a physical barrier maintaining the liquid–vapor interface at the entrance to the pores and water molecules evaporate from the hot feed solution to the cold permeate/distillate [1]. Since in the MD process the hydrophobicity of the membrane is the fundamental necessity [2], the polymeric membranes, such as polypropylene (PP), polytetrafluoroethylene (PTFE), polyvinylidene fluoride (PVDF) and polyethylene (PE), are the most commonly used [3,4,5]. In addition, as was pointed out by Ezugbe and Rathilal [6], MD membranes should be characterized by: (i) low resistance to mass transfer in order to enable free flow of mass, (ii) low thermal conductivity to enhance heat maintenance in the system, and (iii) resistance to the wetting phenomenon. Although various configurations of the MD configuration can be applied, the direct contact membrane distillation (DCMD) is the simplest, the most researched, the best known and the most used.

**Figure 1:**
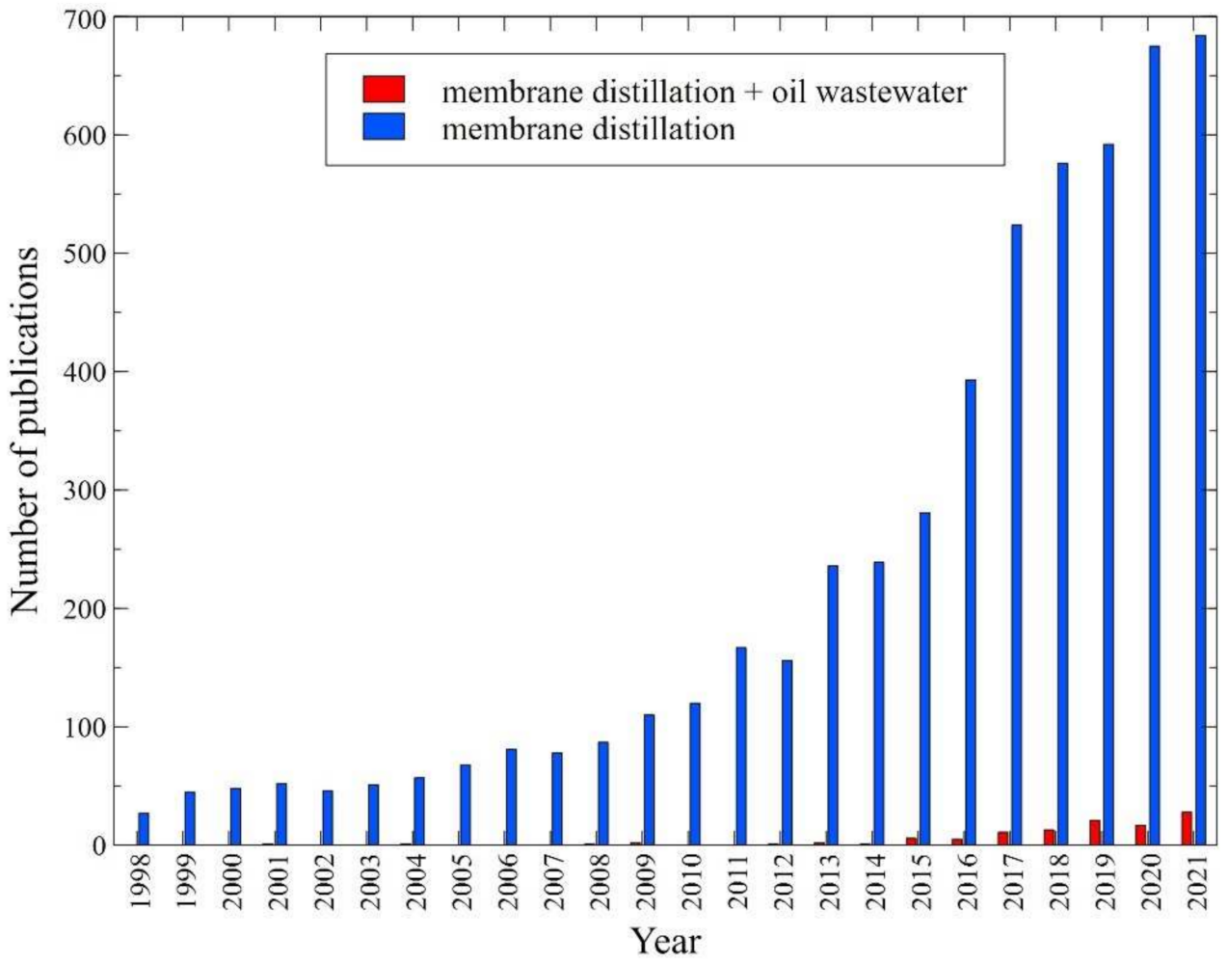
Number of publications focused on the MD process and wastewater treatment. Scopus, data retrieved: 22 February 2022.

Theoretically, the MD process ensures the 100% rejection of non-volatile components. MD technology has a gamut of reported successful applications. It was demonstrated that it can be successfully used for seawater desalination [7,8,9,10,11], brine concentration [12,13,14], concentration of fruit juices [15,16,17] and in the decentralized water/electricity cogeneration systems integrating photovoltaic/thermal (CPV/T) collectors and vacuum multi-effect MD [18]. It should be pointed out that recently, implementation of a multi-stage air gap membrane distillation rig for desalination on-board a marine ship was proposed [19]. Moreover, MD is a promising green treatment process for the treatment of various types of wastewaters, such as radioactive [20,21], textile [22,23] and oily [24,25,26,27]. It should be pointed out that in recent years, the application of MD for wastewater treatment in order to ensure water reuse has gained substantial attention. Undoubtedly, this is due to the fact that MD is considered a cleaner process than conventional methods [28]. The literature search for “membrane distillation” in the Scopus database indicated a strong research interest in the MD process. Indeed, more than 5690 articles were published from 1998 to February 2022. Figure 1 presents the trend of publication in the Scopus database using the following keywords “membrane distillation” and “membrane distillation + oil wastewater”, limiting the research papers. It can be observed that numerous MD studies have been performed on the MD process and its implementation for the treatment of wastewater with significant growth in the number of publications during the last 10 years. Nevertheless, it should be pointed out that although attempts have been made with significant research papers over the past decade, comparatively few studies exist that specifically address the application of the MD process for oily wastewater treatment (Figure 1). This finding is in line with the conclusion presented in [29,30], indicating that studies focused on understanding the MD process used for the treatment of oily wastewater are scarce. Furthermore, to the best of our knowledge, studies on the oily wastewaters treatment by MD membranes are mainly limited to model oil in water and water in oil emulsions. This could be related to the varying nature and complexity of oily wastewaters. Additionally, according to Han et al. [29], it could be due to the poor performance experienced.

There is clear evidence that the major concern with respect to the application of the MD process is related to the fouling and wetting issues. Roughly speaking, membrane fouling is caused by the deposit of foulant on the membrane surface and/or within its pores [31], meanwhile, membrane wetting is defined as liquid contacting with a membrane through intermolecular interaction between the phases of gas, liquid, and solid [32]. As it was pointed out by Guan et al. [33], membrane wetting leads to diffusion of the feed liquid and all dissolved salts across the membrane resulting in a rapid decrease in both the permeate flux and salt rejection. It should be pointed out that there is a vast amount of literature on the fouling and wetting phenomena in the MD. Indeed, the above-mentioned phenomena were summarized in several review articles [32,34,35,36,37,38,39,40,41,42,43,44,45,46].

Generally, factors that have an impact on the fouling and wetting phenomena in the MD technology are classified into three groups: (i) membrane surface properties (e.g., wettability, roughness, surface tension, pore size, surface charge, surface functional group), (ii) operational parameters (e.g., temperature, flow velocity, motion direction, local hydrostatic pressure gradient), and (iii) feed properties (e.g., pH, charge, mole fraction, nature) [32]. Among the above-mentioned parameters, the process parameters are one of the most significant since they may improve the permeate flux by controlling the temperature polarization and concentration polarization phenomena. Notwithstanding, as was pointed out by Julian et al. [3] in a recently published review article, although several studies on MD technology have been conducted, the impact of operational conditions on the process performance is still lacking. Therefore, in the context of research findings presented in the literature, the current work aimed to investigate the impact of the feed temperature and flow velocity on the PP membrane performance used for the long-term DCMD process of real oily saline wastewaters. Moreover, for comparison purposes, experiments were also carried out with model NaCl solutions; the Baltic Seawater and oil in water emulsions. 

## 2. Materials and Methods

**Figure 2:**
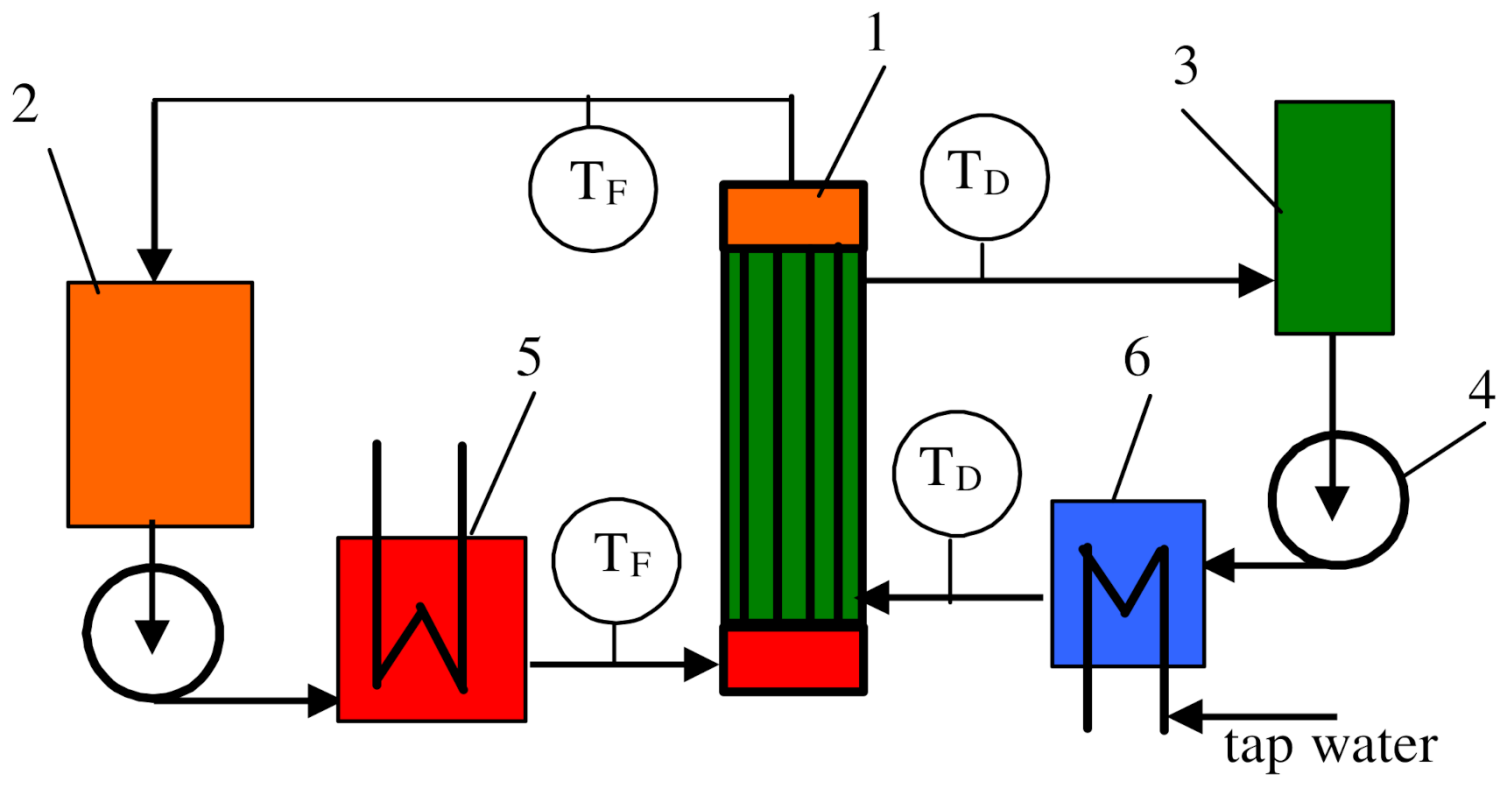
Schematic diagram of the MD installation: 1—capillary module, 2—feed tank, 3—distillate tank, 4—peristaltic pump, 5—heat exchanger, 6—cooler, T—thermometer. F—feed, D—distillate.

In the present study, the MD process was conducted in the direct contact membrane distillation (DCMD) system (Figure 2).

The Accurel PP V8/2 membranes supplied by a commercial manufacturer (Membrana GmbH, Germany) were used. The membranes were characterized by an internal diameter and a wall thickness equal to 5.5 mm and 1.5 mm, respectively, a porosity of approximately 70% and a pore diameter of 0.2 µm. It should be pointed out that in the previous studies [25,47], it was demonstrated that the used membranes show good resistance to separated oily wastewaters. Four membranes (length 7 cm) were mounted inside a glass tube (diameter 20 mm) resulting in an inside membrane area equal to 48 cm^2^. Due to the fouling and scaling phenomena occurring during the conducted research, modules with the new membranes were used in the individual experimental series.

The feed flowed on the lumen side, and the peristaltic pump used allowed to adjust the feed flow velocity (v_F_) in the range from 0.03 to 0.12 m/s. Additionally, the impact of the feed temperature (T_F_) in the range of 323–343 K on the process performance was investigated. The temperature of the distillate flowing on the membrane outer side was equal to 292 +/− 2 K.

Before each series of measurements, the distillate tank was filled with distilled water (0.5 L). In order to determine the obtained permeate flux *J*, after the end of a given test period *t*, the distillate tank was weighed and the permeate flux was calculated from the volume increase (assumed 1 g = 1 mL) for the tested period MD, according to the following equation:

(1) $$J=\frac{\Delta V}{A\cdot t}$$

where *ΔV* is an increase in the volume of distillate at time *t*, *A* is the module area.

After time *t*, the electrical conductivity of the water in the distillate tank was measured. For this purpose, a 6P Ultrameter (Myron L Company, Carlsbad, CA, USA) was used. The meter was calibrated for measurements in NaCl mode using TDS/conductivity standard solution (Myron L Company).

Oily wastewaters (OWW1 and OWW2) produced on ships sailing the Baltic Sea were used as a feed. For comparison purposes, experiments were also carried out with model NaCl solutions and water from the Baltic Sea collected in Pobierowo (Poland). Additionally, MD experimental tests were carried out in which an oil emulsion made of waste machine oil collected in the bilge of the ship’s engine room was added to the above-mentioned solutions. In order to determine the influence of the feed temperature on the scaling phenomenon intensity, the MD processes were carried out at a constant feed concentration by adding to it distilled water in an amount balancing the value of obtained permeate.

The collected oil (density of 0.878 g/mL) was dark brown in color and had a distinct smell of machine oil. An emulsion concentrate was made from 5 mL of oil added to 1 L of distilled water. A small amount of the concentrate was added to the tested solutions, obtaining an oil content in the range of 20–88 mg/L in the feed. The oil content was analyzed using an OCMA 500 oil analyzer manufactured by Horiba (Kyoto, Japan). The analysis of the feed composition was performed using an ion chromatograph (850 Professional IC, Herisau Metrohm, Switzerland) and the membrane samples were observed by scanning electron microscope SEM (Hitachi SU8000, Tokyo, Japan). The membranes’ contact angle and the solutions’ surface tensions were measured with a Sigma 701 microbalance (KSV Instrument Ltd., Helsinki, Finland). The measurements were carried out using the Wilhelmy plate method at ambient temperature (293–294 K). The contact angles of the surfaces of the new membranes were 99.5°. The characteristics of the feed solutions are presented in Table 1.

## 3. Results and Discussion

### 3.1. MD Process of Salt Solution and Oil Emulsion

**Figure 3:**
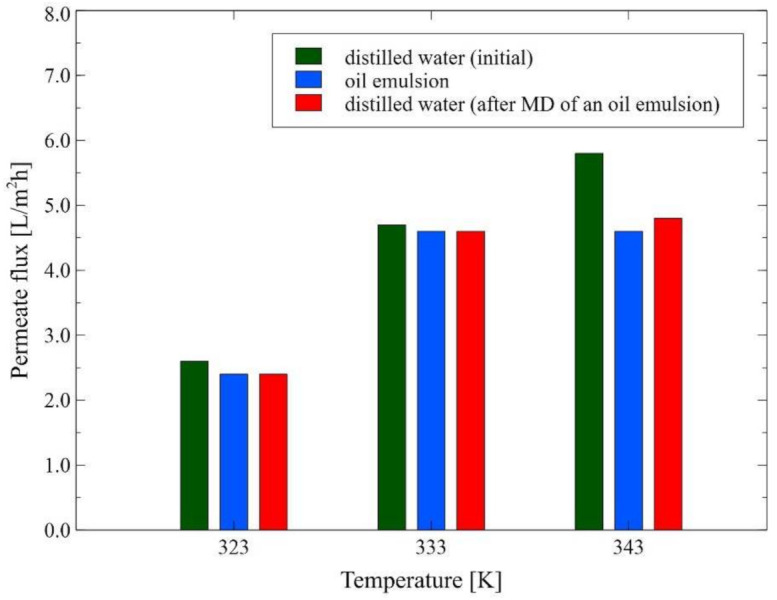
The impact of the feed temperature on the permeate flux. v_F_ = 0.12 m/s. Module M1.

In the present study, before conducting the MD process of real oily wastewaters, model NaCl solution and oil in water emulsions were used as a feed. As established earlier, the driving force in MD is the vapor pressure difference between the two sides of the membrane, hence, it is well recognized that the feed temperature is the primary parameter that has a strong influence on the process performance. In general, the feed temperature in MD is in the range from 308 to 358 K [48] and typical MD processes can be driven by the low-temperature difference (20 K) [49]. In the present study, the impact of feed temperature on the MD process performance was studied over a range of temperatures from 323 K to 343 K with an increment of 10 K, under a constant feed velocity rate of 0.12 m/s. It was observed (Figure 3) that increasing the feed temperature from 323 K to 333 K and subsequently to 343 K, led to an increase in the permeate flux from 2.6 to 4.7 and 5.8 L/m^2^h, respectively. Undoubtedly, a positive correlation between the feed temperature and permeate flux was observed due to enhancement in the process driving force as vapor pressure is exponentially related to temperature, according to the Antoine equation [28,50,51]. In addition, as was pointed out by Ge et al. [52], in the DCMD process, increasing the feed temperature leads to a decrease in the feed viscosity and, thus, to an increase in the Reynolds number, a thinning of the feed boundary layer and an increase in the heat transfer efficiency. It is essential to mention that the results obtained in the present study are consistent with several earlier works [9,24,49,53,54,55,56,57], wherein the increase in the permeate flux when increasing the feed temperature was observed. For instance, Boubakri et al. [57] have investigated the nitrate removal from an aqueous solution by the DCMD process. The authors have demonstrated that increasing the feed temperature from 313 K to 353 K led to an increase in the permeate flux from 0.60 to 4.29 L/m^2^h and from 4.12 to 37.21 L/m^2^h for PP and PVDF membranes, respectively. In turn, in [52], the membrane fouling and wetting phenomenon in a DCMD process applied for an RO brine concentration were studied. It was shown that the relative flux of the hollow-fiber PVDF membrane at 350 K was higher than that at 328 K, irrespective of the type of feed used (pure water, 36.2 g/L NaCl and 46.5 g/L RO brine).

Nonetheless, in the present study, it was demonstrated that adding an oil emulsion (oil concentration in the feed: 30 mg/L) to distilled water (used as a feed) resulted in a significant reduction of the permeate flux. Indeed, the permeate flux during the MD process of an oil emulsion, for the feed temperature of 323 K, 333 K and 343 K was equal to 2.4, 4.6 and 4.6 L/m^2^h, respectively. After 6 h of the process run, the emulsion was removed from the system and the membrane module was fed again with distilled water. As a matter of fact, it was observed (Figure 3) that the initial module performance was not recovered. Indeed, it was found that the final values of the permeate flux during the MD process of the distilled water at the temperature of 323 K, 333 K and 343 K were equal to 2.4, 4.6 and 4.8 L/m^2^h, respectively. Undoubtedly, the obtained results could be attributed to the membrane fouling phenomenon. Indeed, oil contaminations adsorb on the surface of hydrophobic membranes [58], which limits the access of the feed to their pores. Similar fouling behavior was found in [59], where the performance of the PVDF membranes during the DCMD process of crude oil in water emulsion was studied. It was indicated that the hydrophobic–hydrophobic interaction between the feed and the membrane enhanced the attachment of oil to the membrane surface, which finally led to the significant flux decline during two hours of operation.

To be complete, it should be observed that after completion of the test runs shown in Figure 3, the installation was fed with the replaced distilled water and subsequently, the MD process was continued at the feed temperature of 343 K. Consequently, a slight increase in the permeate flux (up to 5 L/m^2^h) was observed. The importance of this point is limited efficiency in removing the oil from the surface of hydrophobic membranes by cleaning them with hot water. In turn, in [24], it was demonstrated that recovering the initial permeate flux of the PP membranes used for the treatment of the oil field-produced water can be achieved by simple washing with deionized water. As recognized in the literature [60], the highly effective membrane regeneration with water can be achieved by membrane modifying that ensures increased hydrophilicity of their surface. It is worth quoting that probably, for this reason, the higher efficiency of the membranes cleaning with water was achieved in the previous study [25], where the oily wastewaters were separated. Indeed, apart from oil fouling, a layer of carbonates and sulfates was formed on the membrane’s surface leading to an increase in the hydrophilicity of the membrane’s surface.

**Figure 4:**
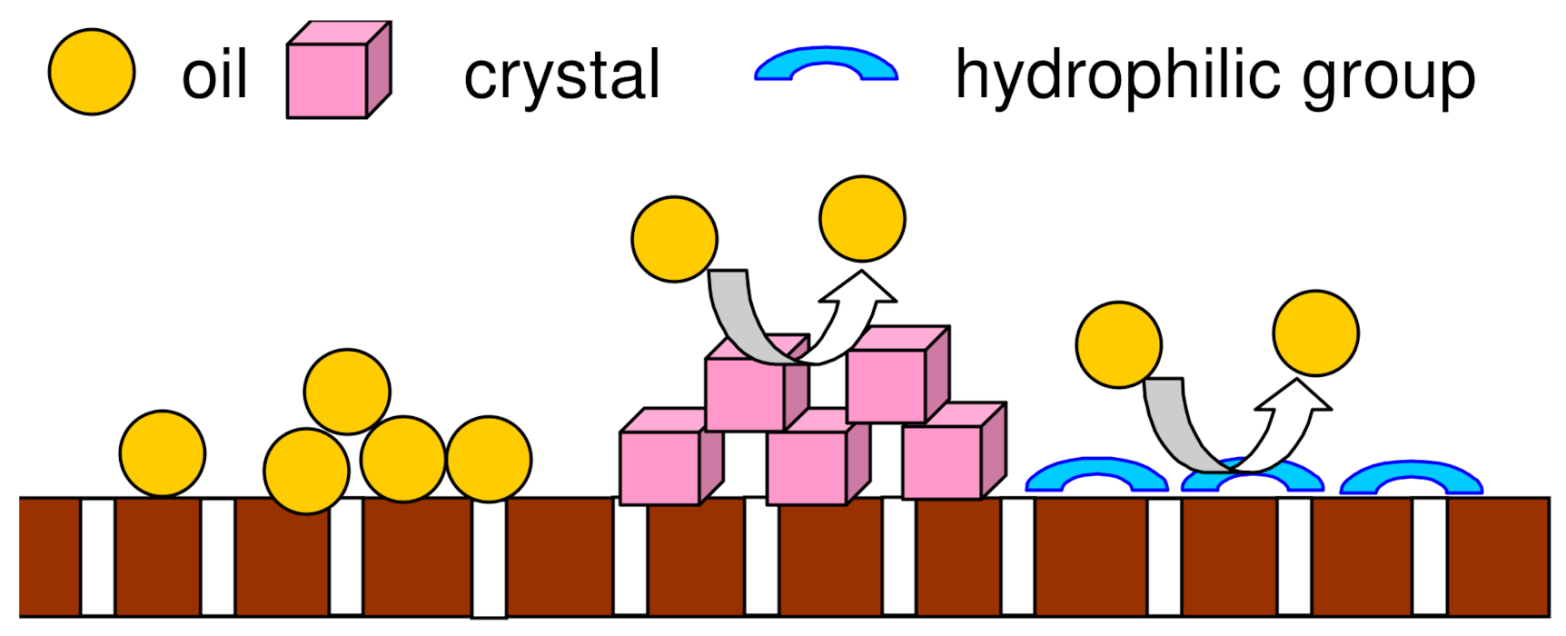
Impact of scaling phenomenon on the oil adsorption on the hydrophobic membrane surface.

The hydrophilization of the membrane surface has a significant impact on the oil fouling phenomenon [27]. Indeed, it is well known that scaling through the formation of a hydrophilic salt layer can reduce oil adsorption. Since the intensity of the changes on the membrane’s surface increases with the time of the MD module operation, the lifespan of the membrane module has a significant impact on the oil fouling, which is schematically shown in Figure 4.

**Figure 5:**
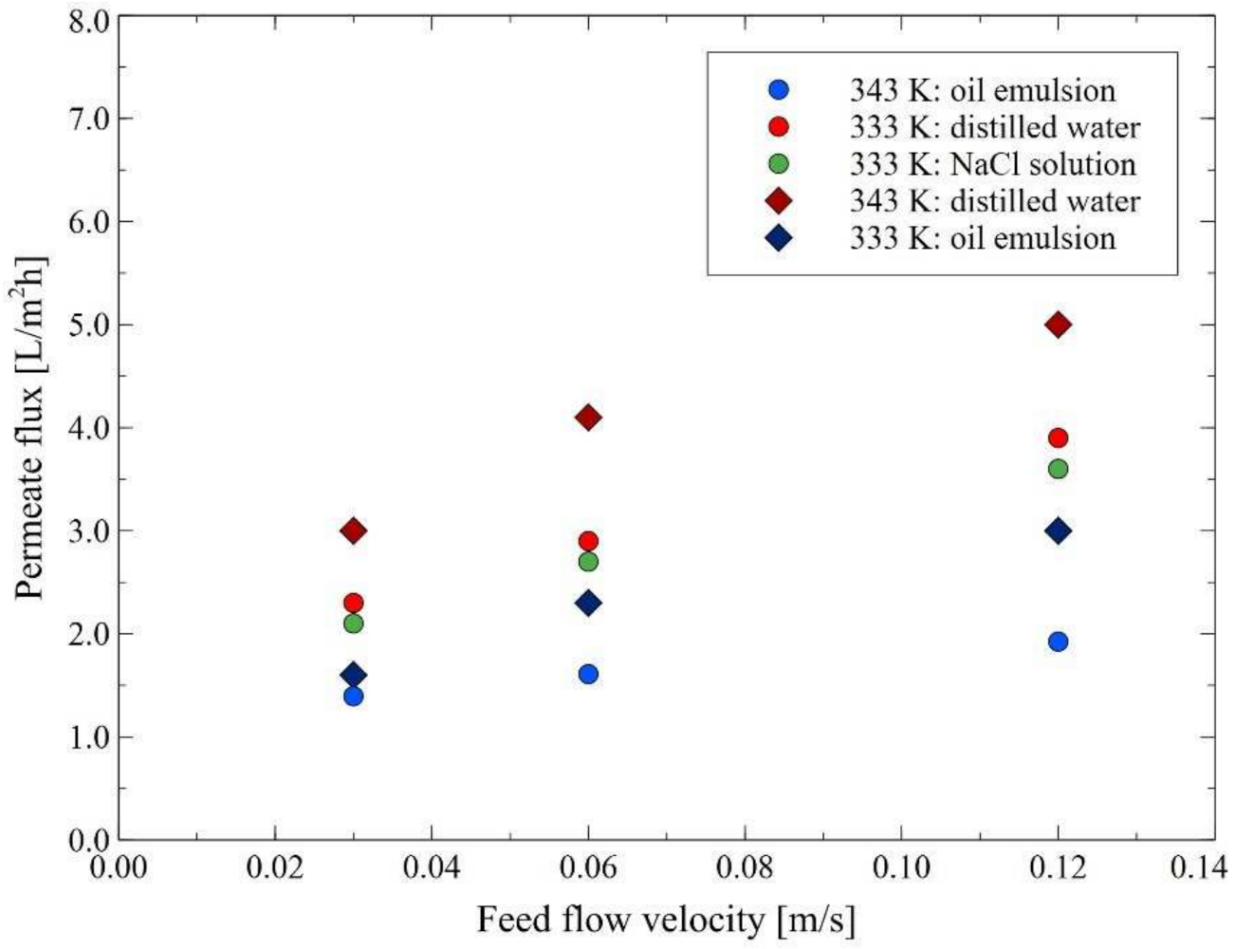
The impact of the feed flow velocity on the permeate flux. Feed: distilled water (T_F_ = 333 K and 343 K), NaCl solution (50 g/L) (333 K), oil emulsion (333 K and 343 K). Module M1.

In the present study, the effect of the feed flow velocity on the MD process performance was evaluated for various feed solutions: distilled water, NaCl solution (50 g/L) and oil emulsion (42 mg/L). It is evident from Figure 5 that the feed flow velocity has a significant impact on the permeate flux for each feed used. For instance, it was found that for the MD process of distilled water carried out at the temperature of 343 K, the reduction of the feed flow velocity from 0.12 m/s to 0.03 m/s led to the decline in the permeate flux from 5 L/m^2^h to 3 L/m^2^h. In turn, at the feed temperature equal to 333 K, the flux reduction from 3.9 L/m^2^h to 2.3 L/m^2^h was observed. With regards to the NaCl solution (333 K), the permeate flux was equal to 2.1; 2.7 and 3.6 L/m^2^h at the feed flow velocity equal to 0.3; 0.6 and 0.12 m/s, respectively. The approximately linearly reduction in the process performance resulted from the phenomenon of temperature polarization, leading the evaporation surface temperature to be lower than that measured in the bulk, which reduces the value of vapor pressure. However, it is well known that polarization phenomena can be reduced by increasing the turbulence of the feed flow [57]. More specifically, high feed flow rates lead to changing the fluid dynamics by increasing the Reynolds number which, in turn, may lead to both a reduction in the thickness of the boundary layer and an increase in the heat transfer from the bulk of the feed stream towards the membrane. Consequently, a reduction in the effects of temperature and concentration polarization can then be achieved [38]. The obtained results were compared with those presented in the available literature. It should be pointed out that the above-mentioned observations are in agreement with those presented in several previous studies, where it was shown that increasing the feed flow rate is beneficial to the increase in the performance of the MD process of various types of feed, such as pure water [33,52], seawater [9], fruit juice [53], highly concentrated solutions of NaCl [33,52,56], KCl, MgCl_2_ and MgSO_4_ [33], NaNO_3_ solutions [57], radioactive wastewater [54], oilfield produced water [49] and carbonate solutions [55]. For instance, Zuo et al. [9] have investigated the permeate flux during the MD process of seawater as a function of the feed flow rate for the polyethylene membrane. The authors have demonstrated that increasing the feed flow rate from 30 L/h to 90 L/h led to an increase in the permeation flux by 85%. To be complete, it should be mentioned that according to Julian et al. [53], changing the feed flow rate is the simplest method as it allows for the reduction of the phenomena of temperature and concentration polarizations. On the other hand, the high flow rate may result in [51]: (i) increased energy consumption, (ii) exceeding the liquid entry pressure of the hydrophobic membrane and consequently a reduction in the membrane selectivity, and (iii) physical damage to the membrane’s active layer. Methods allowing for overcoming the above-mentioned limitations related to the use of high flow rates were discussed in detail by Anvari et al. [51].

Moreover, in the present study, it was demonstrated that the presence of salt (50 g/L of NaCl) in the feed led to a decrease in the permeate flux by about 7% (Figure 5). Obviously, this observation is mainly attributed to the decrease in the water vapor pressure [33]. This finding is in line with results presented in [24], where the MD process with the use of a flat-sheet PP microporous membrane was investigated. In the above-mentioned study, a reduction in water flux for synthetic NaCl solution (135 ppm) compared to deionized water was observed. Indeed, the authors have demonstrated that the average decline in the flux was equal to about 49.3%, 28.3% and 23.5% at the temperature of 313 K, 333 K and 353 K, respectively.

It is essential to mention that at the beginning of the measurement series, significant drops in the process performance were observed after feeding the module with an oil emulsion. Indeed, for the MD process conducted under the feed temperature of 343 K and 333 K, the permeate flux decreased about twice, i.e., from 3 L/m^2^h to 1.6 L/m^2^h and 1.9 L/m^2^h to 1.4 L/m^2^h, respectively. Importantly, the above-mentioned values of the permeate flux are much lower than those presented in Figure 3. This result is related to the higher oil concentration (43–46 mg/L) and longer contact time of the membranes with the emulsion.

**Figure 6:**
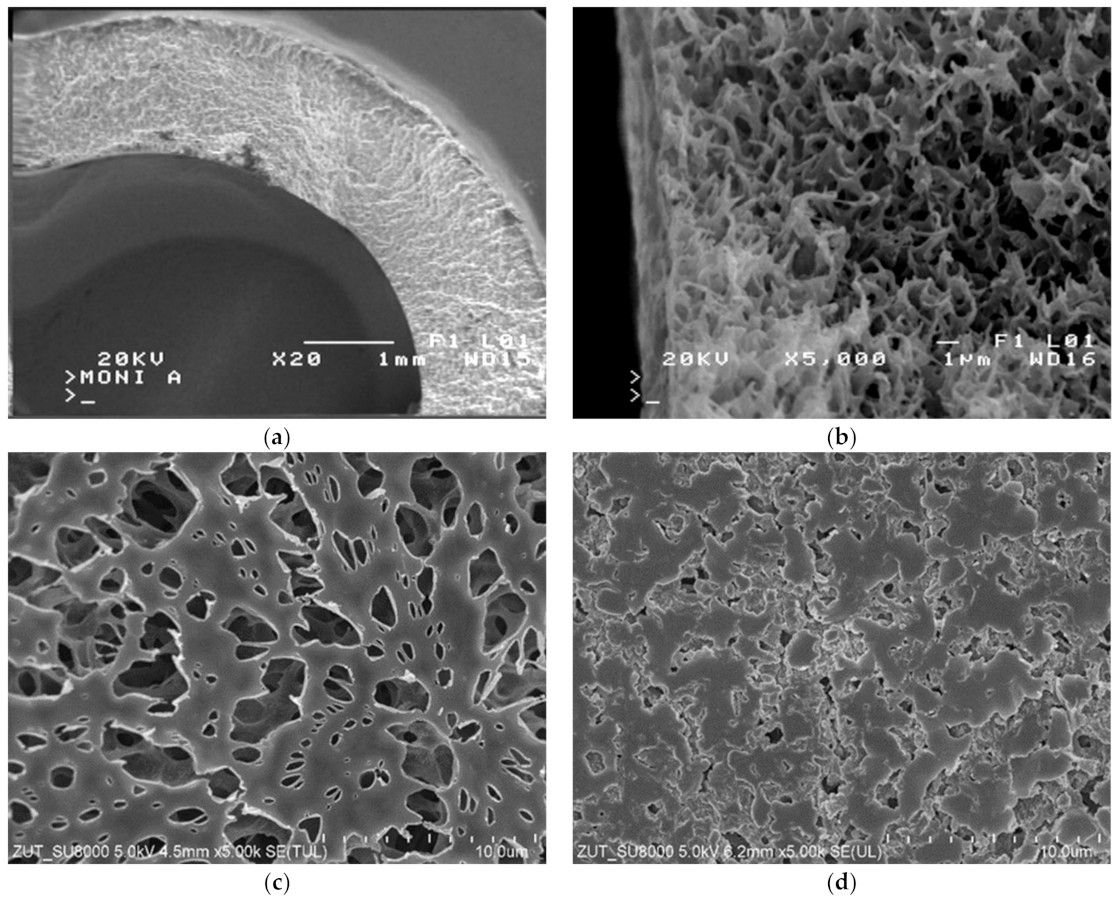
SEM images of Accurel PP V8/2 membrane: (**a**) membrane cross-section; (**b**) pore structure inside membrane wall; (**c**) internal membrane surface of virgin membrane; (**d**) membrane after separation of oil–water emulsion.

The SEM images of the membrane samples mounted in the M1 module are shown in Figure 6. The used membranes have a symmetrical spongy structure inside the wall (Figure 6a,b). The surface porosity (Figure 6c) differed from that observed inside the walls. It was determined that the surface of the membranes (M1 module) after the MD process of oil in water emulsion was more compact and less porous than that of the virgin membrane. Interestingly, SEM-EDX analysis of the membranes’ surfaces revealed the presence of only carbon and chromium, which were sputtered on the membranes for performing the SEM tests. Based on the above findings, it can be concluded that the observed reduction in the membrane surface porosity was due to the oil adsorption, which confirms the pore-blocking mechanism shown in Figure 4 (left side).

### 3.2. MD Process of the Baltic Seawater

**Figure 7:**
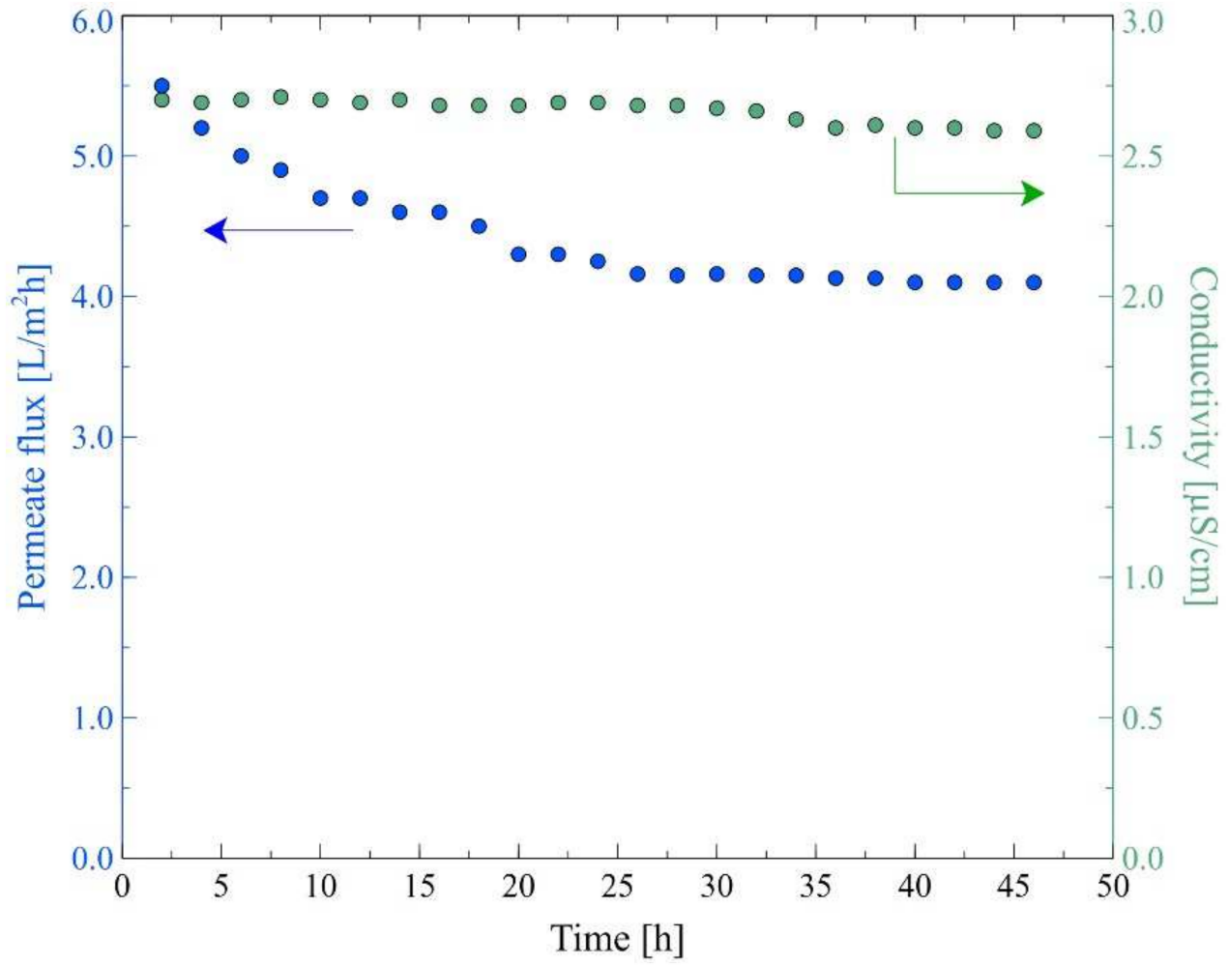
Changes in the permeate flux and distillate conductivity. Feed: NaCl solution (6 g/L), T_F_ = 343 K, v_F_ = 0.12 m/s. Module M2.

The main aim of the present study was to investigate the application of the MD process to the separation of bilge water. The wastewaters used in the experiments, apart from oil pollution, contained the Baltic Seawater, which, due to its low salinity (below 7 g of salt/L), is classified as brackish water. The Baltic Seawater also contained other components (Table 1) that, during the MD process, form deposits of CaCO_3_ and CaSO_4_ on the membrane’s surface [61]. An important point that should be observed is that the Accurel PP membranes used in the current study have repeatedly proven excellent suitability for the long-term MD processes [25,27,47,61]. Nonetheless, it was observed that in the initial period of the process run (50–100 h), the module performance slightly decreased. Hence, as was pointed out in [47], in order to analyze the fouling and scaling phenomena, it is recommended to initially stabilize the operation of the module with a feed that does not cause deposit formation on the membrane surface. For this purpose, in the present work, experimental investigations with the use of the M2 module were started by feeding the system with a solution containing 6 g/L of NaCl. As shown in Figure 7, the most significant decline in the module performance during the first 25 h of the process run was observed. Indeed, it was observed that the permeate flux decreased from 5.5 L/m^2^h to 4.3 L/m^2^h. Subsequently, it was stabilized at a value of about 4 L/m^2^h. Furthermore, it was found that during the process run, the value of distillate conductivity slightly decreased from 2.7 μS/cm to 2.6 μS/cm. These results clearly indicate that the membranes used in the present study were resistant to wetting and, consequently, the feed was not leaking through the membrane’s pores.

**Figure 8:**
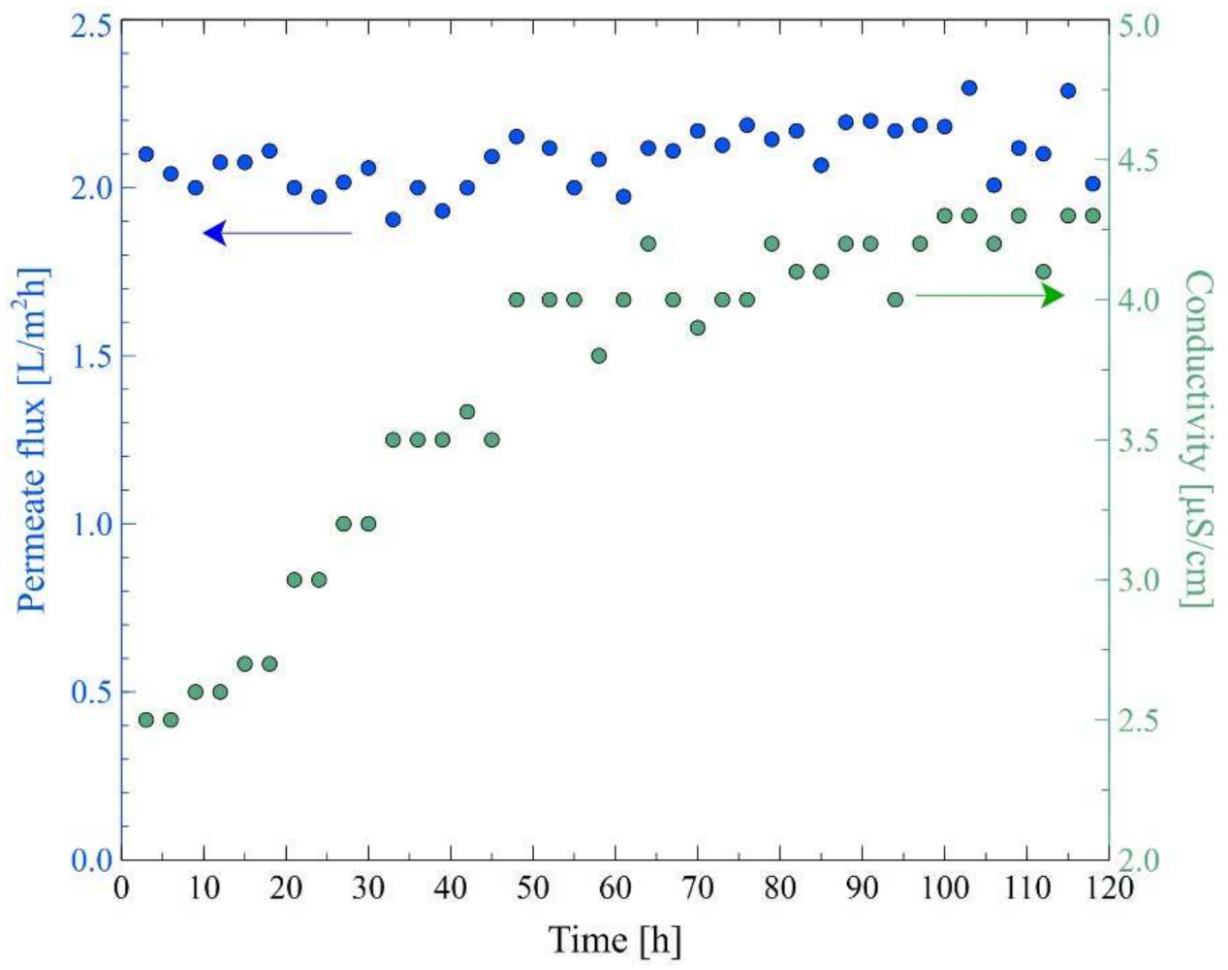
Changes in the permeate flux and distillate conductivity. Feed: Baltic Seawater, T_F_ = 323 K, v_F_ = 0.12 m/s. Module M2.

As established earlier, the formation of deposits on the membranes leads to limitation of both water flow and heat transport to the evaporation surface, hence, a decrease in the MD process performance can be observed. Worthy of note, in the previous work [61], it was demonstrated that conducting the MD process at the low feed temperature allows for reducing the formation of deposits by the components of the Baltic water. The results presented in Figure 8 substantiated that notion. Indeed, during the MD process (323 K) of the Baltic Seawater, a stable permeate flux was observed. Since the intensity of the scaling phenomenon increases with the increase in the feed concentration, in this part of the research, the experiments were carried out for a constant concentration of the feed by adding distilled water in an amount balancing the value of obtained permeate.

**Figure 9:**
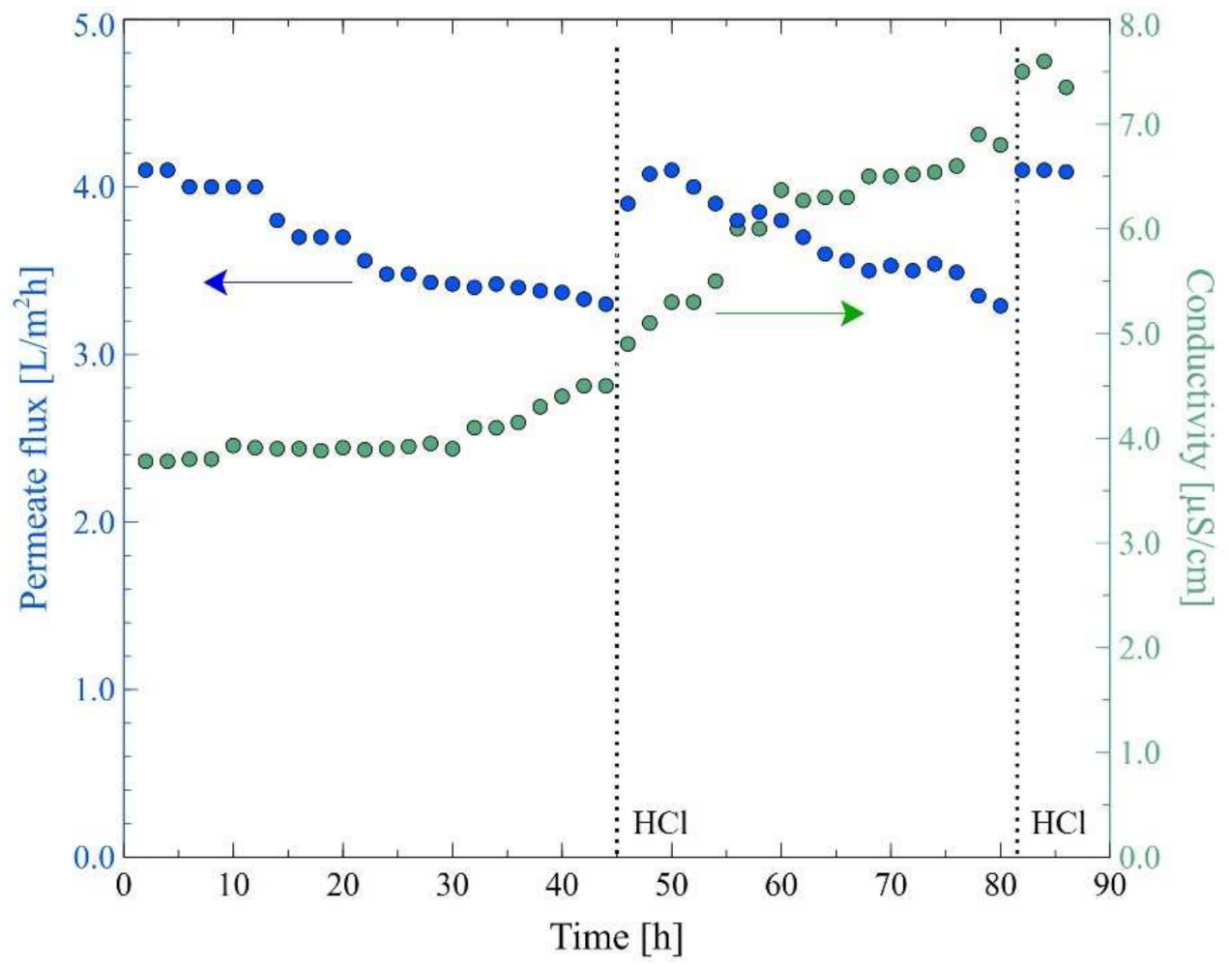
Changes in the permeate flux and distillate conductivity. Feed: the Baltic Seawater, T_F_ = 343 K, v_F_ = 0.12 m/s. Module M2. A 45 h and 82 h of the MD process run—the module rinsing with 3% HCl solution.

**Figure 10:**
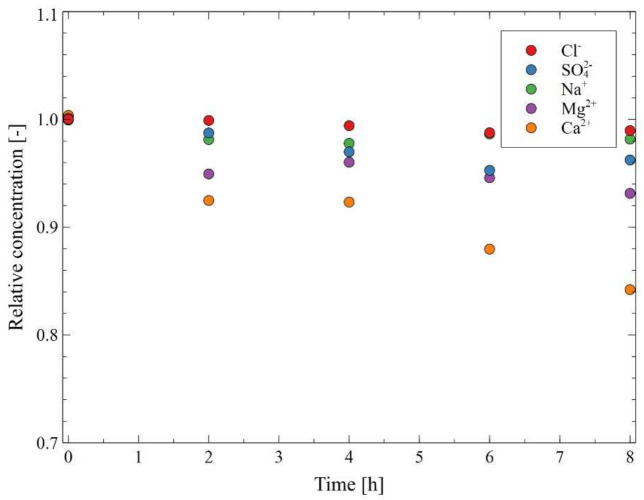
Changes in the composition of the Baltic Seawater during the MD process. T_F_ = 343 K, v_F_ = 0.12 m/s. Module M2.

In the subsequent step of the MD studies, the experiments were carried out for the higher feed temperature (343 K). As expected, after a few hours of the MD run, a systematic decrease in the permeate flux was observed (Figure 9). It must be stressed that increasing the temperature of the feed disturbs the solution equilibrium, which may cause the deposition of carbonates. This conclusion is consistent with the analysis of the changes in the Baltic Seawater composition during the MD process conducted under the temperature of 343 K (Figure 10). Despite maintaining a constant feed volume (a concentration factor of 1 as a result of adding distilled water), the ion concentration changed, especially a decrease in the concentration of calcium in the tested water was observed. Importantly, it was found that the formation of deposits caused pores wetting, which, in turn, led to a slight increase in the distillate conductivity to 4.5 μS/cm (Figure 9). Additionally, it was determined that performing the procedure of membranes rinsing with 3% HCl solution (45 h and 82 h of the process run) allowed to increase the permeate flux to about 4.1 L/m^2^h. On the other hand, it led to an increase in the value of distillate conductivity. At the end of the process run, distillate conductivity of about 7.5 μS/cm was observed. It should be pointed out that this finding is consistent with earlier results [61,62,63,64], wherein it was demonstrated that the rinsing of MD membranes with HCl solution is an effective method of removing carbonates allowing to restore the performance of the initial module. This result plays a significant role in maintaining the stable and reliable performance of the MD modules used in the long-term processes since, as was pointed out by Warsinger et al. [45], calcium carbonate is the most common scale in thermal desalination systems. Notwithstanding, it should be observed that acid dissolution of deposits may result in increased wettability of the membrane’s surface.

**Figure 11:**
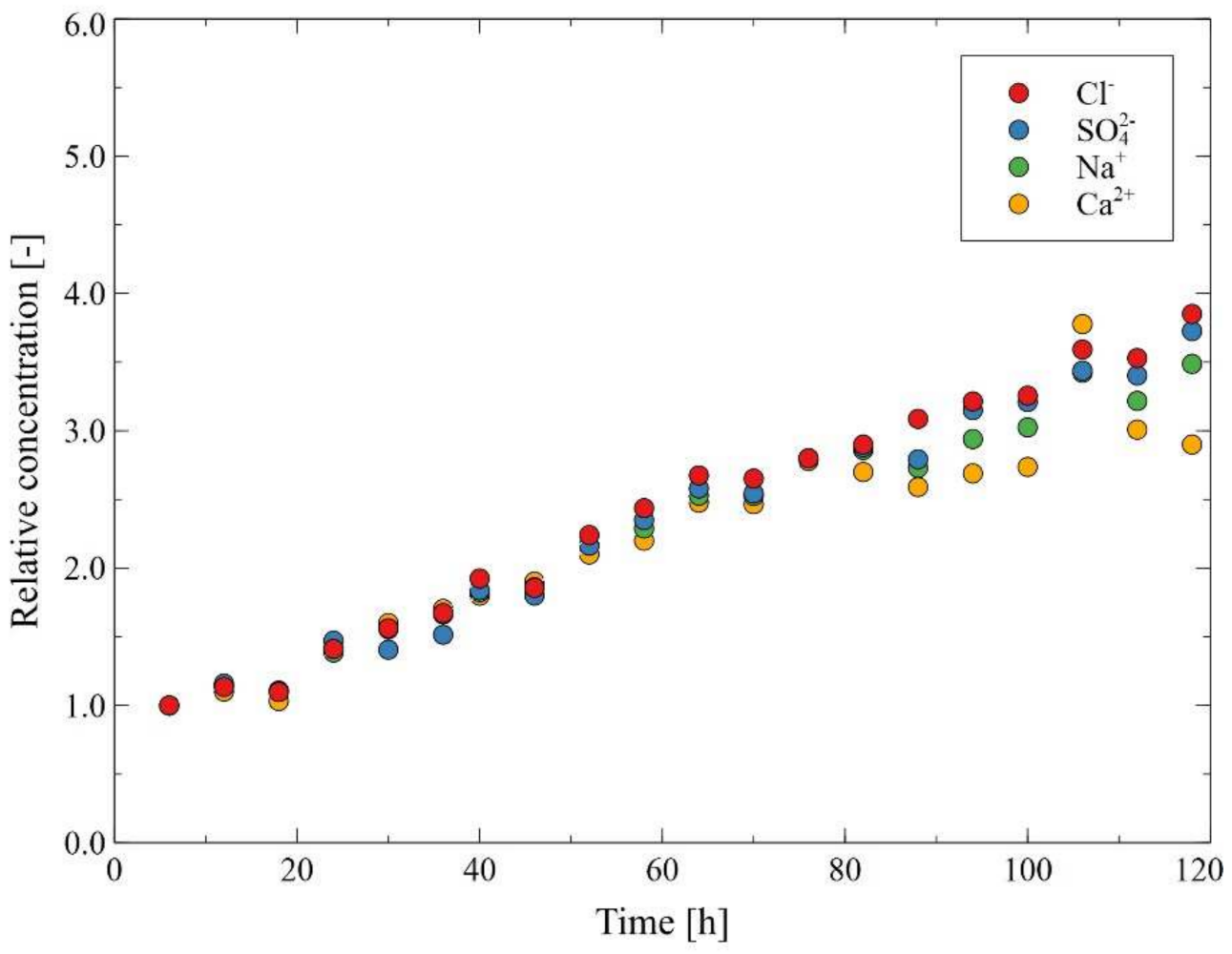
Changes in the composition of the feed during desalination of the Baltic Seawater. T_F_ = 343 K, v_F_ = 0.12 m/s. Module M2.

**Figure 12:**
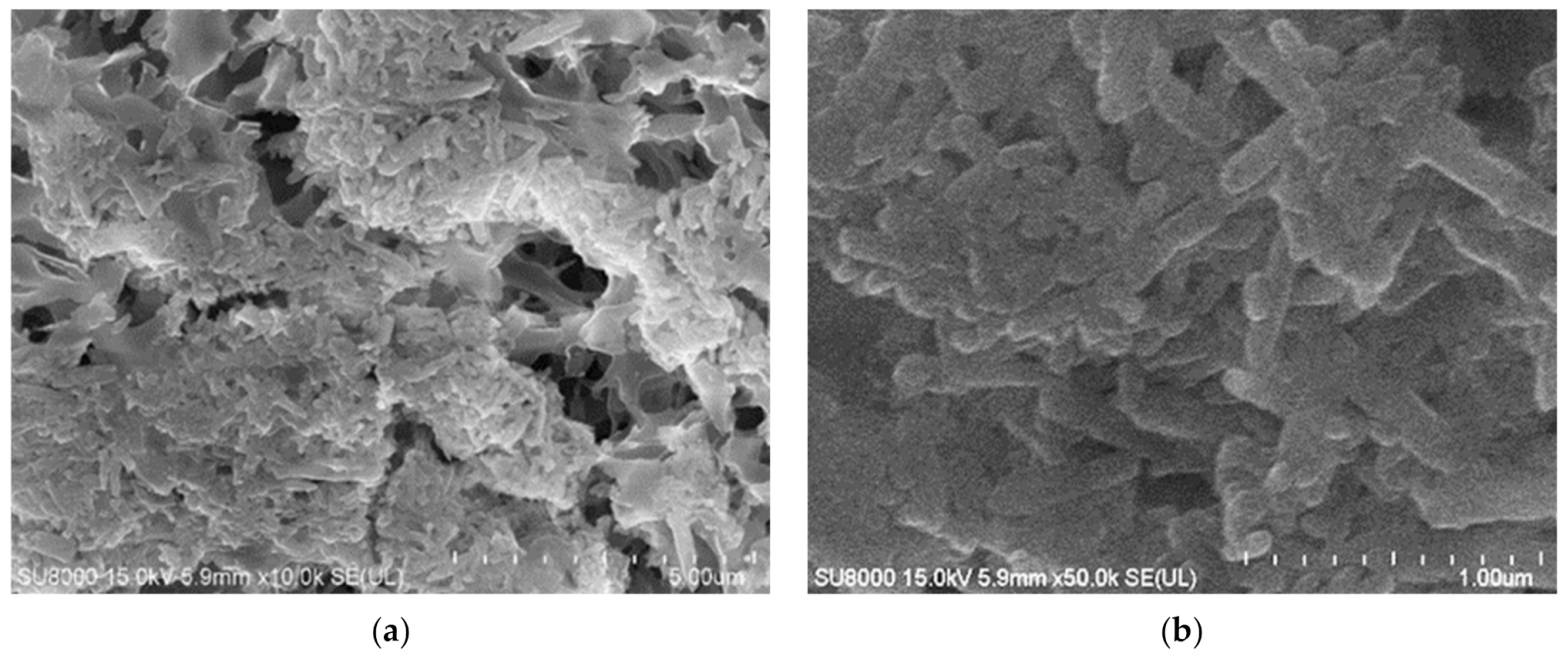
SEM images of: (**a**) Membrane surface covered by deposit formed during desalination process of the Baltic Seawater; (**b**) Deposit layer magnify × 50 k. Module M2.

After the initial formation of the deposit caused by heating the Baltic Seawater in the MD installation, in the next stage of the research, the Baltic Seawater used in the previous process run (Figure 9 and Figure 10) was used in the concentration process. As a consequence, its volume decreased four-fold, which corresponded to the water recovery rate equal to 75%. The results presented in Figure 11 clearly indicate that the carbonate precipitation phenomenon occurred. This observation is consistent with the results of the XRD analyses presented in the previously published study [61]. It should be pointed out that after 120 h of a desalinization process run, the distillate conductivity increased to 12 μS/cm, which proved that the used membranes retained their non-wettability. In turn, the permeate flux decreased to 3 L/m^2^h, which constituted 73% of its initial value. The salt concentration in the feed increased to about 25 g/L, which led to a decrease in the vapor pressure and, consequently, the process driving force decreased. The SEM analysis indicated that scaling was an additional reason for the decline in the process performance. Indeed, it was found that a significant part of the membrane’s surface was covered with a thin layer of the deposit (Figure 12). The SEM-EDS analysis showed the presence of C, O, Ca and small amounts of Mg, Na and Cl.

### 3.3. MD Process of Baltic Seawater Contaminated with Oil

**Figure 13:**
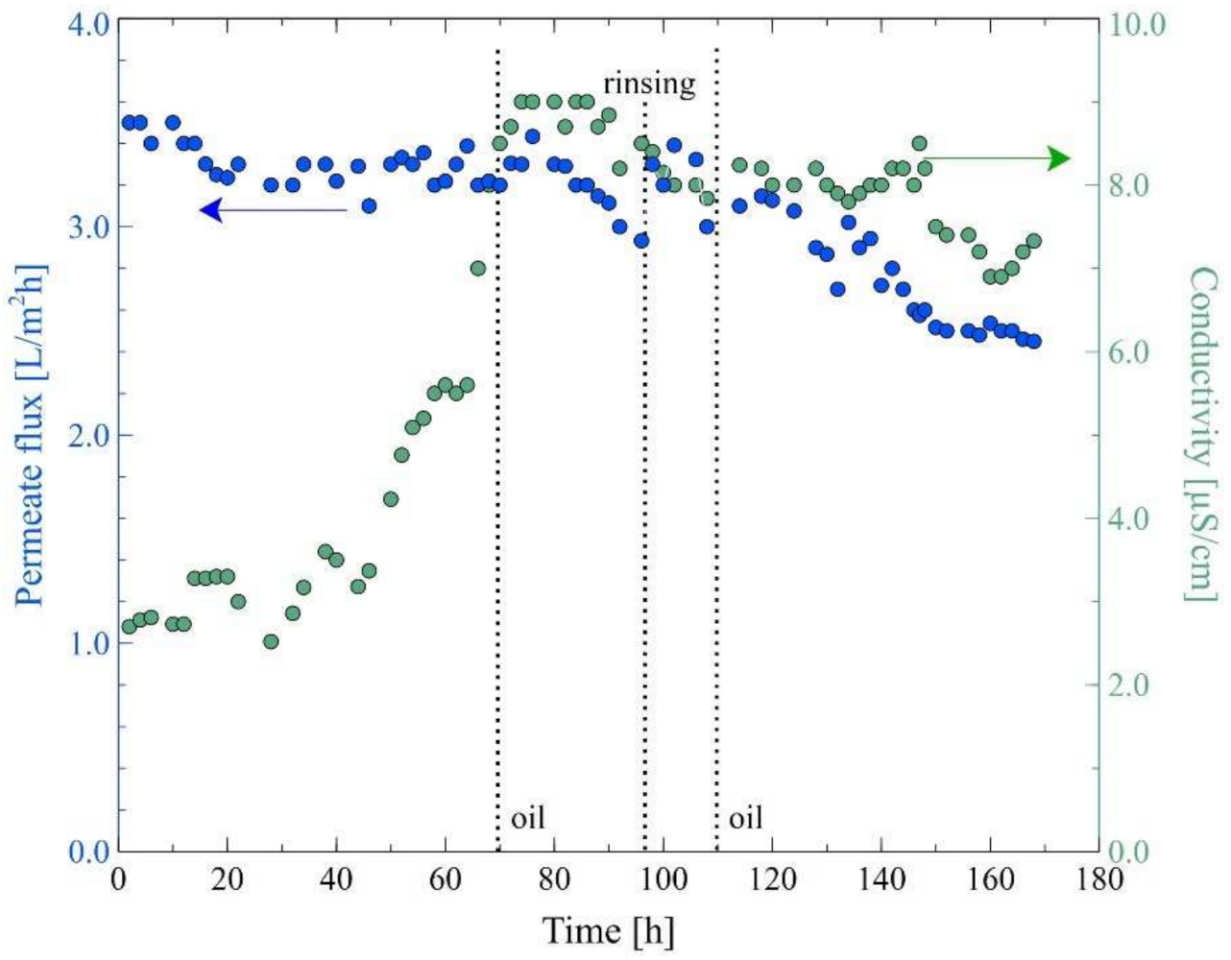
Changes in the permeate flux and distillate conductivity. Feed: Baltic Seawater contaminated with oil, T_F_ = 343 K, v_F_ = 0.12 m/s. Module M3. A 70 h and 110 h of the MD process run—adding oil to the feed. At 95 h—module rinsing by hot water.

Overall, the adsorption of oil on the membrane surface can influence the heterogeneous crystallization of the salt. In the present study, in order to investigate this phenomenon, the Baltic Seawater, with the addition of an oil emulsion (20 mg/L), was used as the feed. It was determined that the surface tension of the prepared solution was equal to 48.2 +/− 1.5 mN/m. It is evident from Figure 13 that contamination of the feed with oil resulted in decreased MD process performance compared to that obtained for the pure Baltic Seawater. Indeed, the initial permeate flux was equal to 3.5 L/m^2^h, however, after 20 h of the process run it decreased to about 3.2 L/m^2^h. Importantly, after 70 h of the process run, a new portion of the oil was added to the feed, as a result of which its concentration increased to 52 mg/L, and, consequently, the feed surface tension decreased to 44.2 +/− 0.3 mN/m. Further increasing the oil concentration in the feed led to a decrease in the permeate flux. Indeed, it was observed that the permeate flux was equal to 2.9 L/m^2^h after 95 h of the process run. Subsequently, the module was rinsed several times with hot distilled water (353 K) and the MD process was restarted. As a result, the permeate flux increased to 3.3 L/m^2^h. However, the achieved results were temporary and a slow decline in the process performance was again observed. Moreover, a significant decline in the permeate flux was observed after adding another portion of oil (feed concentration: 88 mg/L) to the feed (110 h). Finally, at the end of the process run (170 h), the permeate flux was equal to 2.45 L/m^2^h.

**Figure 14:**
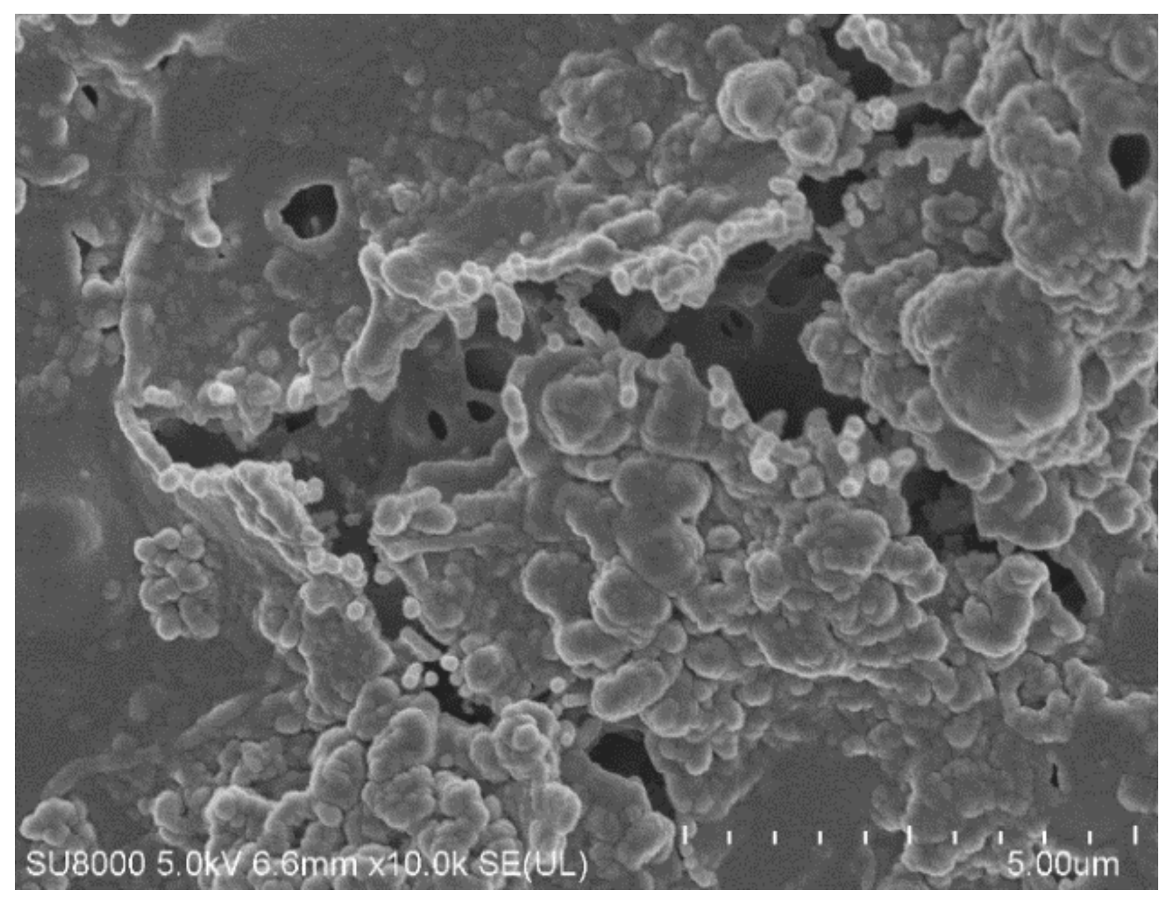
SEM images of membrane surface covered by deposit formed during desalination of Baltic Seawater contaminated with oil. Module M3.

Although the distillate conductivity increased in the initial period of the MD process run, it was stabilized at the level of 7 μS/cm. This result clearly indicates that the decrease in the process performance was due to blocking membranes by deposits formed on their surface. This observation was confirmed by the SEM analysis (Figure 14). Indeed, it was determined that the surface of the membranes was covered with a dense deposit with few lumens. It can be seen that the deposit was more amorphous and was characterized by numerous spherical forms.

### 3.4. MD Process of Oily Wastewaters

**Figure 15:**
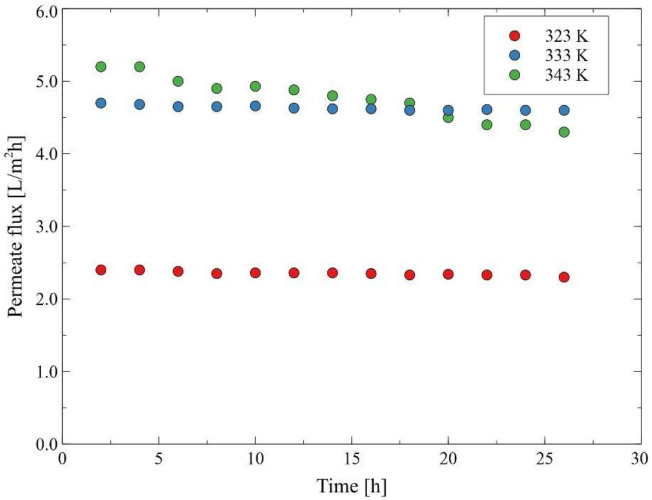
The impact of the feed temperature on the permeate flux. Feed: oily wastewater OWW1, v_F_ = 0.12 m/s. Module M4.

The wastewaters (OWW1 and OWW2) used in the present work contained Baltic Seawater, salt, oils and other pollutants that accumulate in the bilges of ships. In this part, the experimental studies began with the MD process of the OWW1 wastewater. The experiments were conducted consecutively under various values of the feed temperature: 323 K, 333 K and 343 K. The process performance observed for the wastewater was similar to that reported for standard solutions, demonstrated in the previous Sections. Furthermore, roughly speaking, a similar impact of the feed temperature on the MD performance (Figure 15) as in the study of oil emulsion (Figure 3) was observed. However, it was observed that the lower temperature of the feed allows for maintaining the stable permeate flux, which indicates that the scaling phenomenon was limited. Indeed, it was determined that increasing the feed temperature to 343 K caused a slow decrease in the permeate flux, which after 20 h of the process, was lower than that obtained for the temperature of 333 K. Finally, at the end of the process run, the permeate flux was equal to 2.3 L/m^2^h, 4.6 L/m^2^h and 4.3 L/m^2^h at the temperature of 323 K, 333 K and 343 K, respectively. In these series of measurements, distilled water was added to the feed, which allowed for the elimination of the possible influence of the feed concentration that the permeate flux obtained.

**Figure 16:**
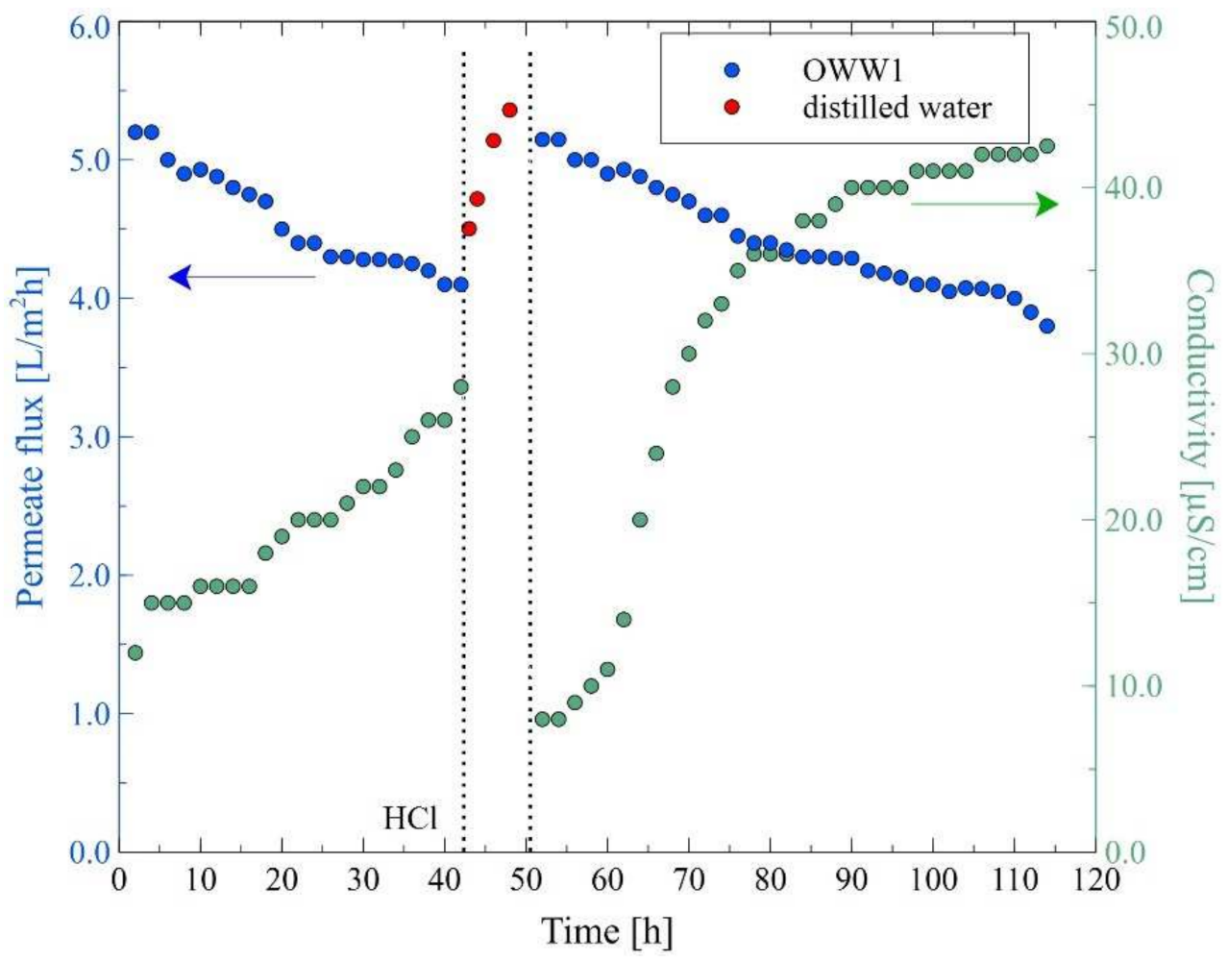
Changes in the permeate flux and distillate conductivity. Feed: oily wastewater OWW1, T_F_ = 343 K, v_F_ = 0.12 m/s. Module M4. At 42 h—module rinsed by 3% HCl, next module flushed by distilled water.

**Figure 17:**
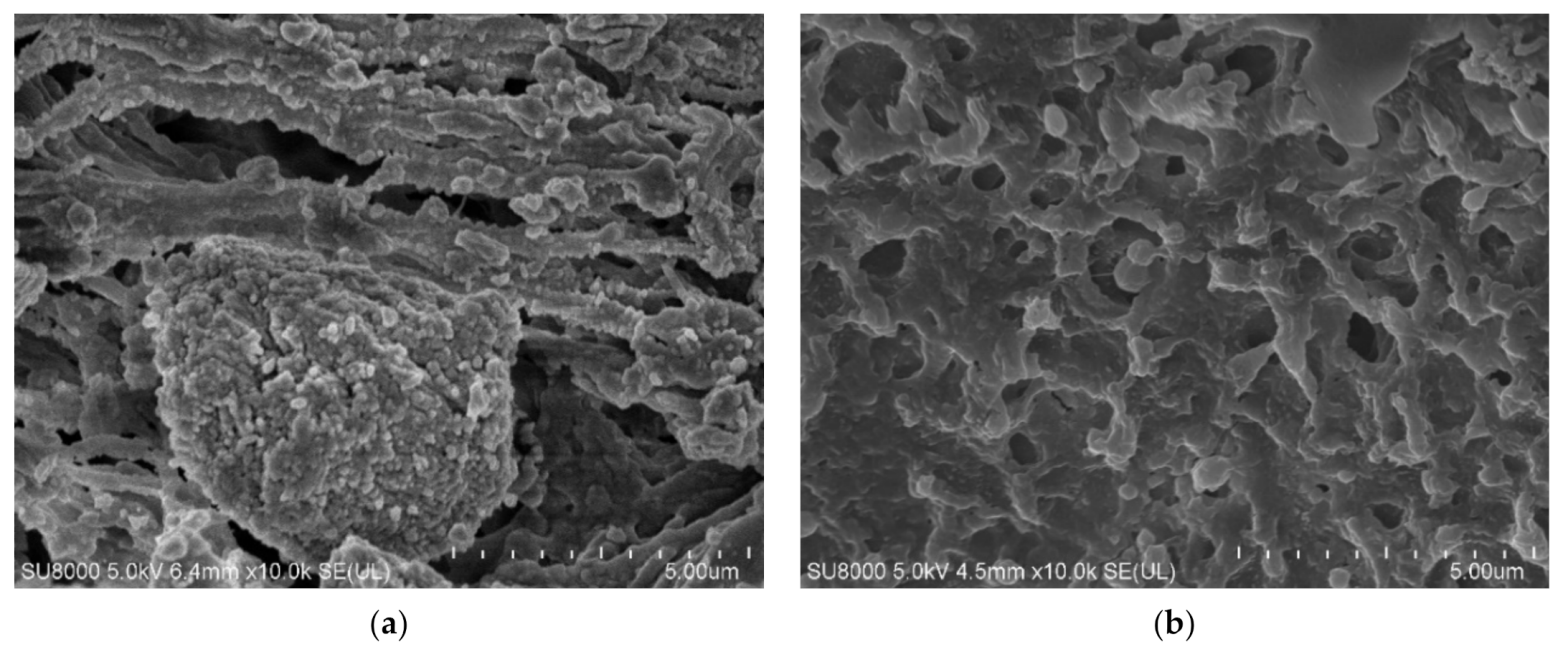
SEM images of membrane surface covered by deposit precipitated from OWW1 wastewater: (**a**) Module M4, T_F_ = 343 K; (**b**) Module M5, T_F_ = 333 K.

As a result of the continuation of the MD process carried out for the feed temperature of 343 K, the permeate flux decreased to 4 L/m^2^h after 42 h of the experiment run (Figure 16). Subsequently, the module was rinsed with 3% HCl solution (15 min) and the MD process was run for several hours (9 h) with distilled water as a feed. The performed membrane washing procedure allowed for an increase in the permeate flux to 5.3 L/m^2^h. After 52 h of module operation, the installation was fed again with the new portion of OWW1 wastewater, which caused a systematic decrease in the process performance. Indeed, it was observed that the values of permeate flux decreased, meanwhile the distillate conductivity increased. At the end of the measurement series (114 h), the permeate flux was equal to 3.8 L/m^2^h, which constituted 72% of its initial value. In turn, the distillate conductivity equal to 43 µS/cm was observed. The performed SEM analysis clearly indicated that the decline in the process performance was caused by the intensive scaling phenomenon (Figure 17a). Indeed, scaling is particularly critical for the MD process since it reduces flux, causes the membrane wetting and may lead to intensification of temperature and concentration polarization on the membrane surface [65]. Yin et al. [66], in the recently published review article, have indicated that the reversibility of inorganic scaling in the MD process has not been well documented in the literature. Notwithstanding, the scaling phenomenon has been reported during the MD process of various feeds, such as oil field-produced water [24], bilge water [25], saline wastewater [26,67] and brine [52]. For instance, in a study conducted by Ge et al. [52], it was determined that during the MD process of brine, different forms of CaSO_4_ crystalline were formed at the PVDF hollow-fiber membrane surface. Indeed, the authors have demonstrated that at a temperature of 350 K, square CaSO_4_ crystals were formed and completely covered the membrane surface, whereas conducting the MD process at a temperature equal to 328 K led to the formation of snowflake crystals which sporadically adhered to the membrane surface.

**Figure 18:**
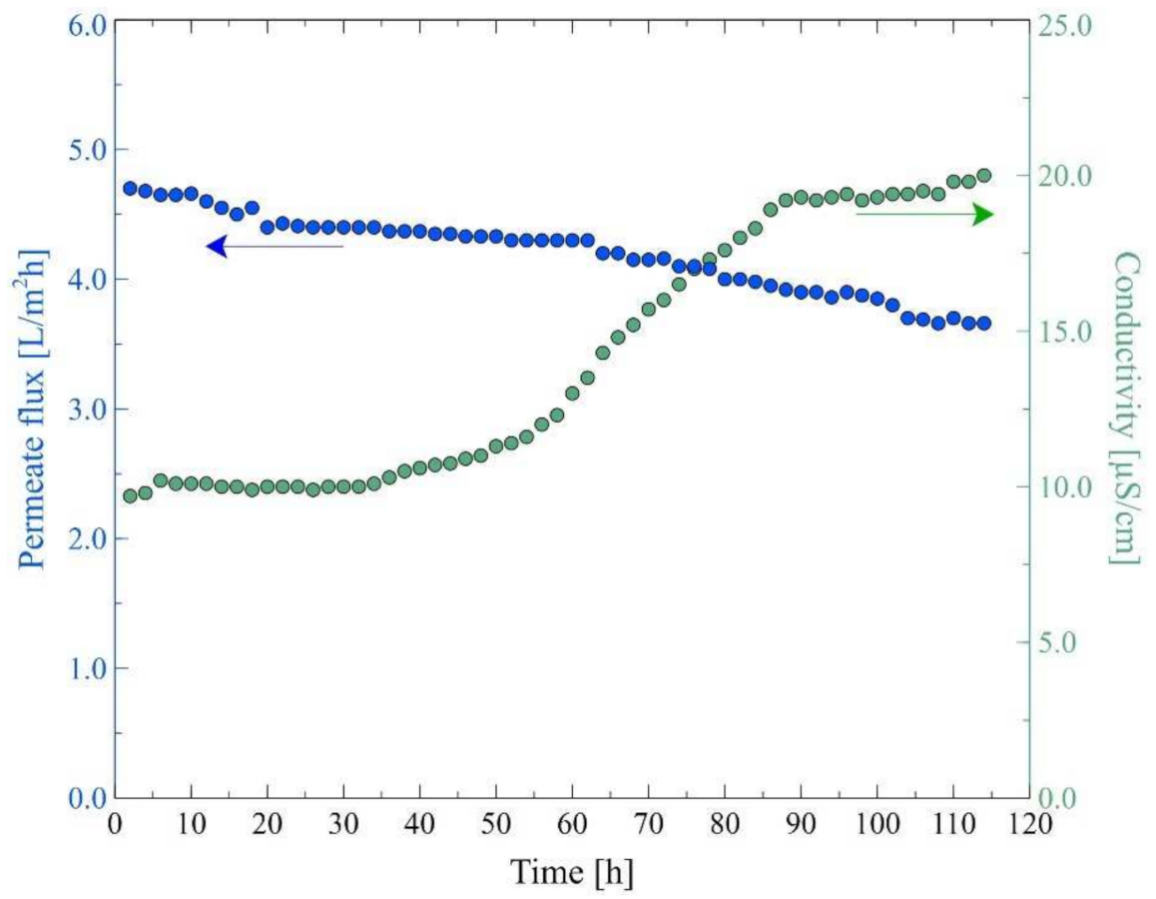
Changes in the permeate flux and distillate conductivity. Feed: oily wastewater OWW1, T_F_ = 333 K, v_F_ = 0.12 m/s. Module M5.

It is well known that increasing the temperature of the feed may allow for increasing the intensity of the scaling phenomenon. For this purpose, in the next stage of the research, the MD process of the OWW1 wastewater was carried out for the feed temperature 10 K lower. As in the previous measurement series (Figure 16), distilled water was added to the feed in order to eliminate the impact of the feed concentration on the permeate flux. It was shown (Figure 18) that conducting the MD process at the feed temperature of 333 K allowed to obtain a more stable permeate flux, which for the first 50 h of the process run was almost constant at the level of 4.6 L/m^2^h. However, in the following hours, the process performance decreased and after 114 h, the permeate flux was equal to 3.86 L/m^2^h (82% of the initial value). Notably, the results of the SEM observations showed that although performing the MD process at the feed temperature of 333 K led to a much lower intensity of the membrane contamination, the entire surface of the membrane was covered with a deposit (Figure 17b).

**Figure 19:**
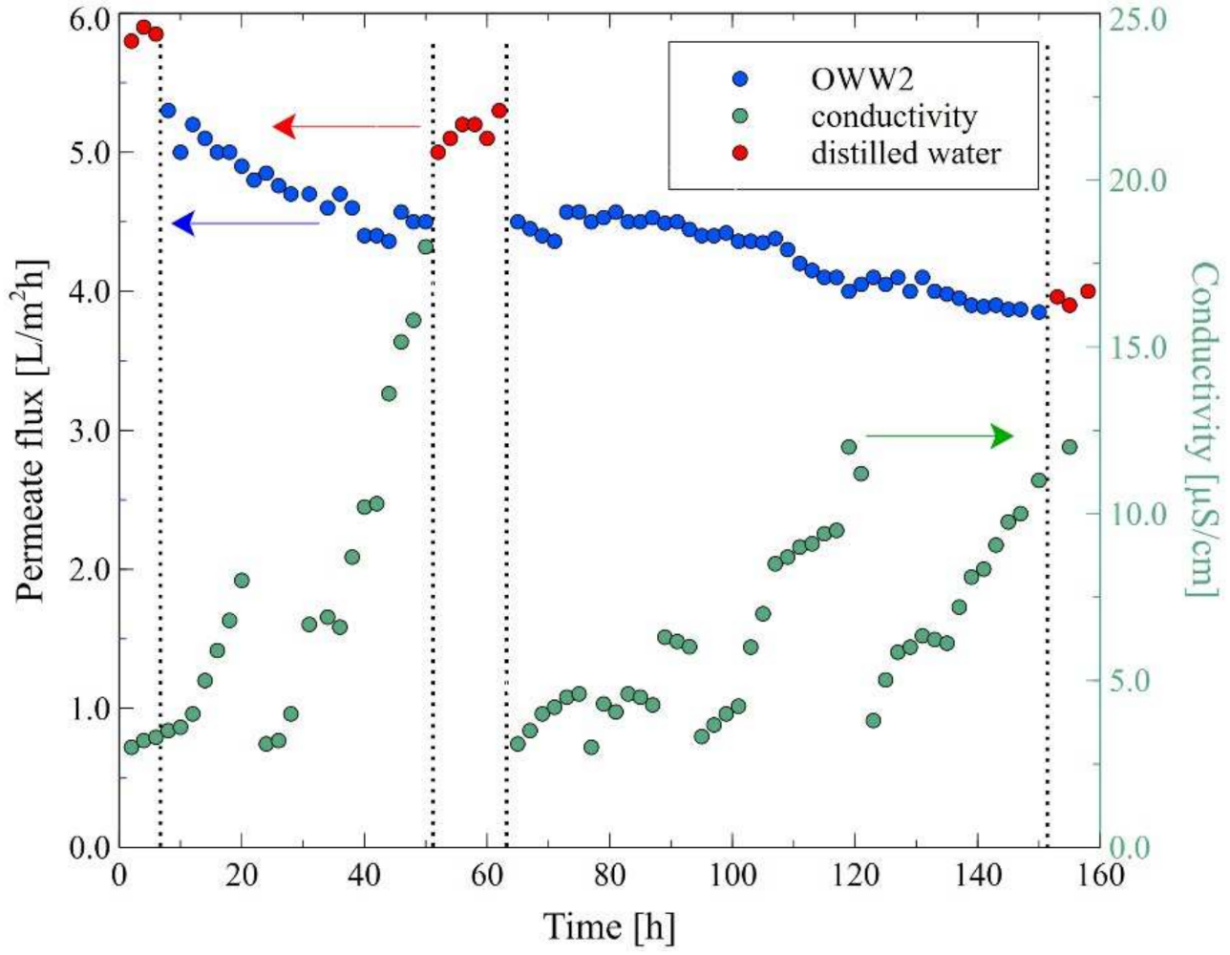
Changes in the permeate flux and distillate conductivity. Feed: oily wastewater OWW2, T_F_ = 343 K, v_F_ = 0.12 m/s. Module M6.

**Figure 20:**
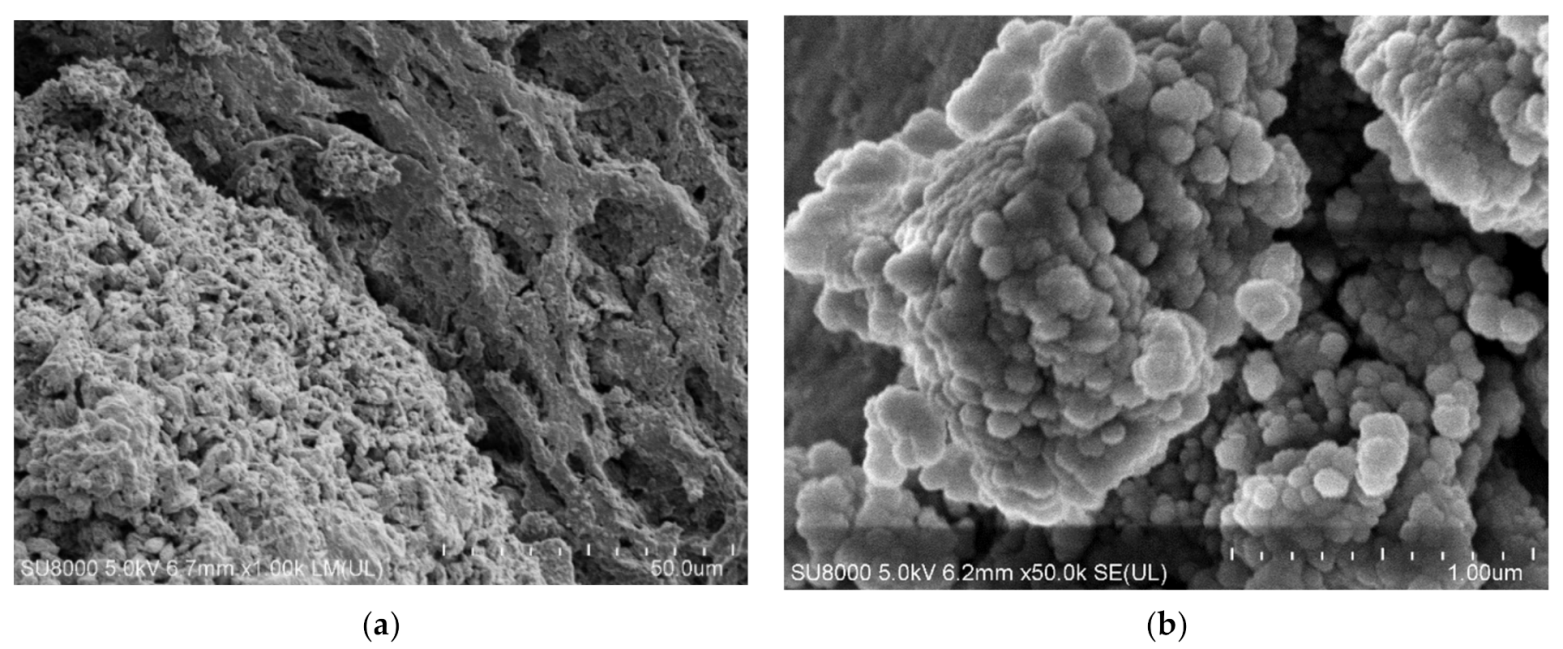
SEM images of: (**a**) Membrane surface covered by deposit precipitated from OWW2 wastewater; (**b**) Deposit layer magnify × 50 k. Module M6.

In the next stage of the research presented, the MD process of the OWW2 wastewater was examined. It was observed during the experimental investigation that the permeate flux was systematically decreasing and the distillate conductivity increased (Figure 19), which indicated the formation of the deposits on the membrane’s surface. As shown in Table 1, the OWW2 was characterized by a higher concentration of components compared to the OWW1. Therefore, during the treatment of the OWW2 wastewater, a more intensive scaling phenomenon was expected. Surprisingly, comparing the results of experiment runs carried out under the same operational parameters (T_F_ = 343 K and v_F_ = 0.12 m/s) for the wastewaters OWW1 (Figure 16) and OWW2 (Figure 19) allowed us to indicate that the obtained performances for both feeds were similar. Undoubtedly, it was due to the fact that despite significant scaling phenomenon occurring during the MD process of the OWW2 wastewater (Figure 20), the deposit structure, similar to that previously reported (Figure 17), was not compact, which allowed the feed to flow to the membrane’s surface.

The SEM-EDX analysis of the deposit composition showed the presence of Ca, C, O, as well as smaller amounts of S, Mg, Na and Cl. This finding is similar to that obtained by Al-Salmi et al. [24], who investigated the application of the DCMD process for the treatment of oil field-produced water. Indeed, in the above-mentioned study, the EDS analysis associated with SEM has demonstrated that the used PP membrane surface was mainly covered by salts containing the following ions: Na^+^, Mg^2+^, Ca^2+^ and Cl^−^. Surprisingly, the results obtained in the present study demonstrated that the shape of the deposits formed by components of oily wastewaters and the Baltic Seawater was significantly different. Indeed, on the surface of PP membranes used for the treatment of oily wastewaters, instead of the sharp-edged forms characteristic for crystals (Figure 12), spherical agglomerates were observed (Figure 20b). Rounded forms were also previously observed on the surface of membranes used for the MD process of the Baltic Seawater contaminated with oil (Figure 14). Therefore, these results offer crucial evidence that the oily components contaminating the feed build into the deposit structure, influencing its shape.

**Figure 21:**
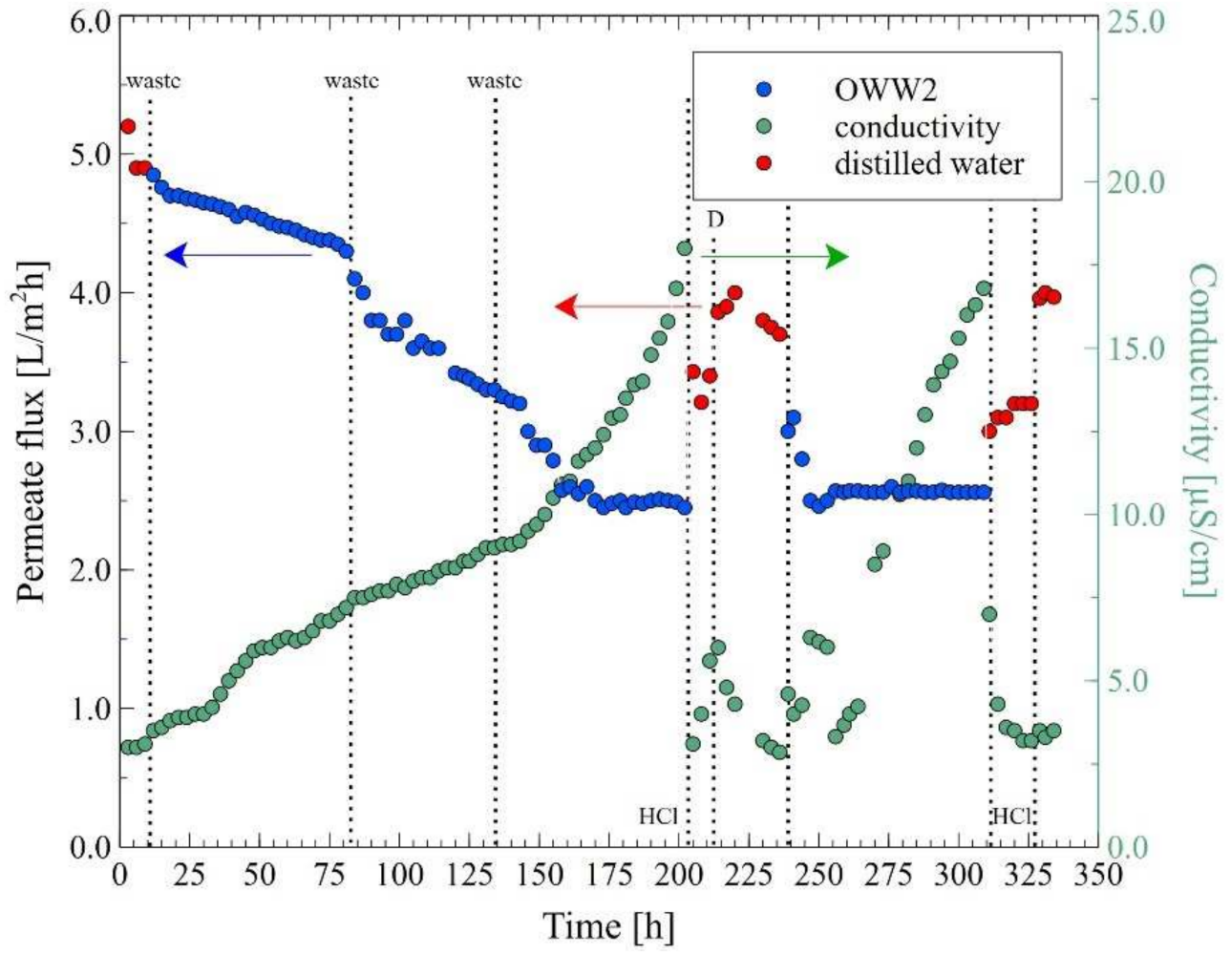
Changes in the permeate flux and distillate conductivity in the MD process with periodic washing of the membranes. Feed: oily wastewater OWW2, T_F_ = 343 K, v_F_ = 0.12 m/s. Module M7. At 10, 85, 135 and 240 h—a new portion of OWW2. Point D—membrane drying.

In the final step of the research, the MD process with the procedure of membranes cleaning was investigated. For this purpose, the MD process of OWW2 wastewater was repeated with the cleaning of the M7 module. Firstly, the membranes were contaminated by replacing the wastewater three times (10, 85 and 135 h). In Figure 21 it can be seen that the most significant decline in the permeate flux during the first hours of the process operation was observed. As it was demonstrated earlier, it was caused by the deposition of carbonates resulting from increases in the feed temperature. It was determined that after 210 h of the process run, the maximum module performance observed for distilled water decreased from 5.2 L/m^2^h to 3.8 L/m^2^h. Subsequently, the module was rinsed with 3% HCl, which allowed to increase the permeate flux to 4 L/m^2^h. 

**Figure 22:**
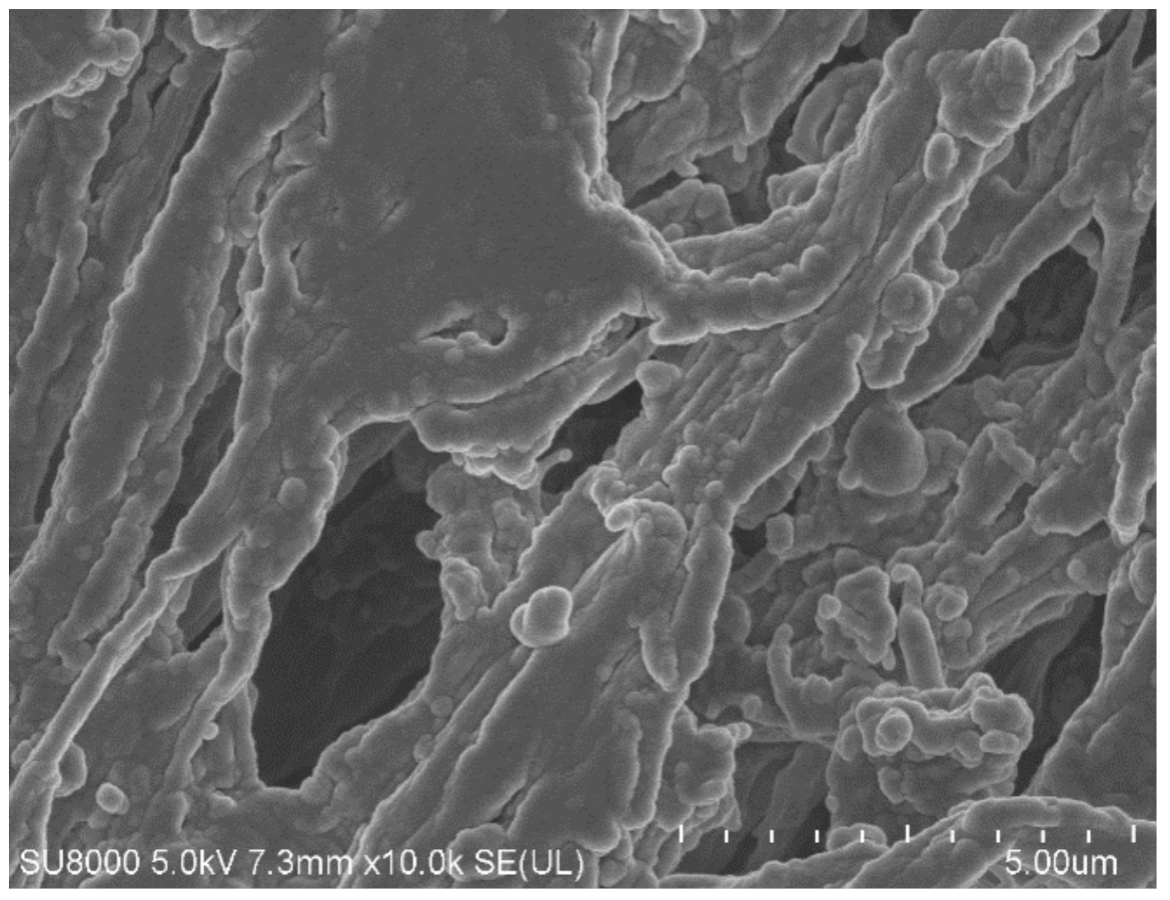
SEM images of membrane surface after module M7 rinsing with 3% HCl solution.

It is well known that in addition to the scaling phenomenon, the decrease in MD process performance is influenced by the membrane pores wetting. Therefore, generally, removing the carbonate deposit and drying the membranes allows for the recovery of their maximum performance. It should be pointed out that in this research, although the membranes were dried, the obtained permeate flux decreased from 4 L/m^2^h to 3.8 L/m^2^h. This noteworthy result indicates that washing the membranes with acid did not remove all impurities. Consequently, the residues caused a more intense blocking of the pores of the membrane. The presence of small amounts of amorphous deposits on the membrane’s surface after washing them with HCl solution was confirmed by the SEM analysis (Figure 22).

The MD process of the OWW2 wastewater effluent was continued and it was reported that the permeate flux decreased again to the value of 2.6 L/m^2^h and it remained stable for another 60 h of the process run. Replacing the wastewater with distilled water allowed for an increase in the flux to 3.2 L/m^2^h, which, after washing the membranes with 3% HCl, again increased to 4 L/m^2^h. The key highlight is, therefore, that performing a cyclic rinsing of the module with a dilute acid solution could be an effective way to maintain a satisfactory MD module performance during the separation of oily wastewaters when a deposit containing CaCO_3_ is deposited on the membrane’s surface.

## 4. Conclusions

The present work aimed at investigating the impact of the operational parameters (feed temperature and flow velocity) on the commercial PP membranes performance used for the DCMD process of oily saline wastewaters. For comparison purposes, experiments were also carried out with model NaCl solutions, the Baltic Seawater and oil in water emulsions. Undoubtedly, based on the results obtained from this work, it can be stated that the PP membranes have excellent potential for application in the sustainable long-term treatment of oily wastewaters generated on ships, such as bilge waters. It was observed during the experimental investigation that for each feed solution used, both temperature and flow velocity have a significant impact on the MD process performance. Indeed, it was observed that an increase in the feed temperature allows for increasing the permeate flux. Notwithstanding, the evidence from this study implies that during the treatment of oily wastewaters the lower temperature ensures a limited membrane scaling phenomenon. Likewise, a higher flow velocity led to a higher flux due to the reduced temperature polarization effect. Furthermore, it was determined that the oily components contaminating the feed build into the deposit structure, influencing its shape. Last but not least, it was found that performing a cyclic rinsing of the module with a 3% HCl solution is an effective method to maintain a stable MD module performance. To sum up, it should be pointed out that the present study has shed the light on improving the MD for the treatment of oily wastewaters, such as bilge water.

## Figures and Tables

**Table 1:** The ion and oil concentration in the oily wastewaters (OWW1 and OWW2) and Baltic Seawater.

Ions (mg/L)	Na^+^	Cl^−^	Mg^2+^	Ca^2+^	K^+^	NO_3_^−^	SO_4_^2−^	Oil (mg/L)
OWW1	2159	3254	318	465	79	110	688	25
OWW2	2775	4338	415	765	116	138	793	42
Baltic Seawater	2297	3189	258	154	83	5.5	478	0

## Data Availability

The data presented in this study are available on request from the corresponding author. The data are not publicly available due to the institutional repository being under construction.
